# PRC2.1 Coordinates Peri‐Nucleolar H3K27me3‐Enriched Heterochromatin Organization and NPM1 Pentamerization to Maintain Nucleolar Integrity

**DOI:** 10.1002/advs.202519359

**Published:** 2026-06-16

**Authors:** Lina Zhu, Wenqin Wang, Shuyun Chen, Gangyi Zhu, Zhen Wu, Yue Gu, Jingwen Xiong, Xu Zhang, Heng Liu, Kyoichi Isono, Youming Zhang, Jingjing Cao, Xiangzhi Li

**Affiliations:** ^1^ Shandong Provincial Key Laboratory of Development and Regeneration, Key Laboratory for Experimental Teratology of Ministry of Education School of Life Sciences Shandong University Qingdao Shandong China; ^2^ School of Health and Life Sciences Qingdao Central Hospital (Breast surgery department) University of Health and Rehabilitation Sciences Qingdao Shandong China; ^3^ NHC Key Laboratory of Otorhinolaryngology Department of Otorhinolaryngology Qilu Hospital of Shandong University Jinan Shandong China; ^4^ Laboratory Animal Center Wakayama Medical University Wakayama Japan; ^5^ State Key Laboratory of Microbial Technology Microbial Technology Institute Shandong University Qingdao Shandong China

**Keywords:** H3K27me3‐enriched heterochromatin, NPM1 pentamerization, nucleolus, phase separation, PRC2.1 (PCL2)

## Abstract

The nucleolus, a membrane‐less organelle within the nucleus, plays a fundamental role in ribosome biogenesis with cellular functions across diverse physiological and pathological states. While its multilayered, liquid‐like architecture is formed by complex interactions between nucleolar proteins and nucleic acids, the regulatory mechanism of nucleoplasmic components on nucleolus remains poorly understood, particularly heterochromatin and its associated proteins. In this study, we revealed that Polycomb repressive complex 2.1 (composed of PRC2 core subunits plus Polycomb‐like (PCL) homolog), associating with H3K27me3‐enriched heterochromatin, accumulates surrounding the nucleolus to form a ring‐like structure. Notably, as a key PRC2.1 component, PCL2 undergoes phase separation, while the intrinsically disordered region (IDR) in PCL2 regulates both the fluidity of the condensates and its interaction with nucleolar protein nucleophosmin (NPM1). PCL2 is a key regulator to facilitate NPM1 pentamerization and interaction among nucleolar components, ensuring the efficient progression of rRNA and protein synthesis. Taken together, our findings reveal that PRC2.1(PCL2) plays a crucial role in maintaining nucleolar integrity and rRNA synthesis, highlighting that PRC2.1(PCL2) is required for cell proliferation.

## Graphical abstract

PRC2.1(PCL2)‐coordinated H3K27me3‐enriched PNH establishes a spatial scaffold crucial for nucleolar integrity. As a crucial coordinator, PCL2 links PRC2.1 to chromatin organization and NPM1 assembly. This network‐based model reveals how chromatin modifications and nucleolar components cooperatively maintain nucleolar architecture, revealing novel insights into the structural roles of PRC2.1 beyond its traditional transcriptional repression.

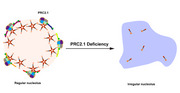

## 1 Introduction

The nucleolus, a membrane‐less organelle in the nucleus, is involved in various cellular processes, such as ribosome biogenesis [1], signal recognition particle formation [2], stress response [3, 4, 5], cell cycle regulation [5], viral replication [6], and stem cell differentiation [7]. The nucleolus is partitioned into three distinct compartments/layers‐the fibrillar centre (FC), the dense fibrillar component (DFC), and the granular component (GC)‐responsible for the successive maturation of ribosomal subunit precursors [8]. Specifically, synthesis of pre‐rRNA by RNA polymerase I occurs at the interface between the FC and the DFC, whereas maturation and assembly with ribonucleoproteins (RNPs) occur in the DFC and GC, respectively [1]. Nucleolar compartmentalization is dynamic, forming sub‐phases that enable a spatial organization of distinct nucleolar components [9]. Multivalent interactions between DNA/RNA and nucleolar proteins form multiphase liquid condensates with a pH gradient [10]. A ternary mixture comprising the GC protein nucleophosmin 1 (NPM1), FC protein fibrillarin (FBL), and a generic rRNA is sufficient to form a multilayered droplet in vitro, similar to the nucleolar sub‐phases [9]. NPM1, a key component of the GC layer, forms NPM1 pentamers critical for GC structural integrity and morphology [11, 12, 13]. Additionally, the interaction between NPM1 and other nucleolar components has been proposed to regulate liquid‐liquid phase separation (LLPS), controlling the directional migration of pre‐ribosomal particles from FC/DFC to GC and ultimately releasing them into the nucleoplasm [13, 14]. Some human diseases are associated with mutations in genes encoding various nucleolus‐associated proteins, such as Werner syndrome [15], Bloom syndrome [16], and cancer [17]. Several mutations on ribosome‐associated proteins induce dysregulated nucleolar organization and impair overall integrity [18]. The nucleolus undergoes dynamic changes through the cell cycle, including disassembly and reassembly [19]. During interphase, the nucleolus expresses hyperactive RNA polymerase I, which supports ribosomal RNA (rRNA) synthesis and facilitates the assembly of rRNP into ribosomes, thereby meeting cellular demands for high levels of RNA transcription and translation. The nucleolus disassembles when cells enter mitosis, and transcription shuts down [20, 21], indicating a highly coordinated relationship between nucleolar morphology and the cell cycle.

Peri‐nucleolar heterochromatin (PNH) is highly condensed chromatin surrounding the nucleolus and relies on epigenetic modifications [20]. Heterochromatin proteins HP1α/β associate with post‐translation modifications of histones in the nucleolar periphery, play critical roles in regulating nucleolar architecture, and function in mouse embryonic stem cells [21], suggesting heterochromatin modification is highly involved in nucleolar morphology and function. Some histone modifications facilitate a stepwise establishment of heterochromatin [22] and generally lead to inhibition of gene transcription, such as H3K9me3 and H3K27me3 [23, 24, 25], which are catalyzed by Suppressor of variegation 3–9 homolog 1/2 (SUV39H1/2) and Polycomb repressive complex 2 (PRC2), respectively [25, 26, 27]. Heterochromatin is divided into constitutive heterochromatin and facultative heterochromatin. Constitutive heterochromatin contains abundant H3K9me3, packages hyper‐inactive pericentromeric satellite repeat sequences, and is involved in nucleolar organizing region (NOR) formation [21]. However, the role of facultative heterochromatin enriched with H3K27me3 in regulating nucleolar morphology remains to be elucidated. PRC2 is one of the two major Polycomb group complexes (PcG) [27, 28]. Histone methyltransferase ‌enhancer of zeste homolog 2 (EZH2), together with embryonic ectoderm development (EED) and suppressor of zeste 12 (SUZ12), are the core components that catalyse methylation of H3K27. Besides the core components, accessory factors, including Polycomb‐like proteins (PCLs; PCL1/PHF1, PCL2/MTF2, and PCL3/PHF19) in PRC2.1 and AEBP2/JARID2 in PRC2.2, are also involved in complex formation. Mammalian PCLs are homologues of the *Drosophila* Polycomb protein (dPCL) [23, 29]. PCLs possess a Tudor domain, two plant homeodomain fingers (PHDs), an extended homologous (EH) domain, and a chromo‐like domain [26, 30, 31].

In this study, we first analyze the subcellular localization of H3K9me3 and H3K27me3. Large H3K9me3 aggregates predominantly localize in the nucleoplasm, with some near the nucleolus. In contrast, H3K27me3 aggregates encircle the nucleolus, forming a ring‐like structure. Moreover, NPM1, a protein located in the nucleolar outer layer, partially colocalizes with and interacts with components of the PRC2.1 subcomplex, suggesting that PRC2.1, along with H3K27me3‐enriched PNH, regulates nucleolar morphology and function. Depletion of PRC2.1 decreases H3K27me3 and pentameric NPM1 levels, as well as interaction between NPM1 and FBL, causing irregular nucleolar morphology and weakened rRNA synthesis. Current study identified the novel function of PRC2.1 in maintaining nucleolar homeostasis.

## 2 Results

### 2.1 PRC2.1(PCL2) Exhibits a Circular Distribution Surrounding the Nucleolus

**FIGURE 1:**
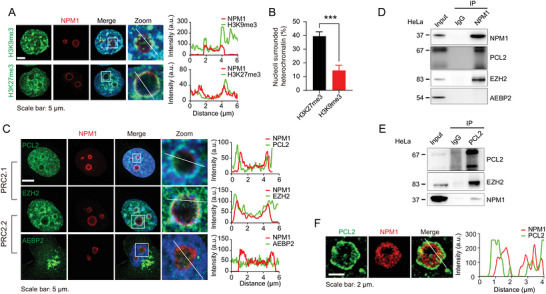
NPM1 colocalizes and interacts with the PRC2.1 subcomplex. (A) Left panels: Subcellular localization of H3K27me3 or H3K9me3 (green), NPM1 (red), and DAPI (blue) in HeLa cells is shown in the representative immunofluorescence (IF) images. Right panels: intensity profiles of NPM1, H3K27me3 and H3K9me3 correspond to the zoomed regions (white rectangle) indicated by white lines crossing the nucleolus in the left panels. *Y*‐axis: intensity, arbitrary units (au); *X*‐axis: distance (µm). (B) Individual nucleoli from triplicate independent experiments in (A) were analyzed. The nucleoli surrounded by ring‐like structure of H3K9me3 or H3K27me3 were counted and presented as a percentage (%) of the total nucleoli in HeLa, SiHa, and CaSki cells (the total nucleoli number, n>200). Statistical analysis was performed with two‐tailed Student's *t*‐test. ^***^, *P* < 0.001. (C) Left panels: Subcellular localization of PRC2 complex subunits (green), NPM1 (red), and DAPI (blue) in HeLa cells is shown in the representative images. The regulatory factor of PRC2.1 subcomplex, PCL2 (upper); the catalytic centre of PRC2 complex, EZH2 (middle); and the regulatory factor of PRC2.2 subcomplex, AEBP2 (bottom), were stained. Right panels: Fluorescence profiles of NPM1 and PRC2 complex subunits from the zoomed area (white rectangle) indicated by white lines crossing the nucleolus in the left panel. (D, E) HeLa cells were lysed and immunoprecipitated using mouse anti‐NPM1 antibody (D), rabbit anti‐PCL2 antibody (E), or IgG of the corresponding species as a control. Immunoblotting was performed to detect NPM1, PCL2, EZH2 and AEBP2. (F) The subcellular localization of exogenous EGFP‐PCL2 and mCherry‐NPM1 in HeLa cells was observed and analyzed.

The nucleus contains highly condensed heterochromatin, which is caused by post‐translational modifications, especially histone methylation [21, 32, 33]. The heterochromatin located at the nucleolar periphery has been demonstrated to contain H3K9me3 and H3K27me3 [32]. H3K9me3, together with its reader HP1, regulates the integrity and function of constitutive heterochromatin in the nucleolus [21]. However, the relationship between H3K27me3‐marked facultative heterochromatin and the nucleolus remains poorly understood. First, we analyzed the subcellular localization of H3K9me3‐ or H3K27me3‐ marked heterochromatin in HeLa, SiHa, and CaSki cells. In these cell lines, both H3K9me3 and H3K27me3 localized in the nucleus, but their distribution differed (Figure 1A, Figure S1A–D). H3K9me3 mainly accumulated in the nucleoplasm, forming granular aggregates (Figure 1A, Figure S1A,C, upper). H3K27me3 exhibited a robust “rim enrichment” pattern, forming an approximately closed circular ring surrounding the NPM1‐marked nucleolus. Both the intensity profiles of H3K27me3 and NPM1 are characteristic of “double‐peak”, confirmed that H3K27me3 accumulates at the nucleolar boundary (Figure 1A, Figure S1A,C, bottom). The encirclement spatial relationship between H3K27me3‐enriched PNH and the nucleolus was further validated in multiple cell types, including primary somatic cells (MEF) and other tumor cell lines (Hepa 1–6, MCF‐7 and MDA‐MB‐231) (Figure S1E). Moreover, the percentage of the nucleolus with a ring‐like structure formed by H3K9me3 or H3K27me3 signal was calculated. Substantial amounts of the nucleoli are encircled by H3K27me3 aggregates (Figure 1A, Figure S1A–D), and 40% of nucleoli contain H3K27me3‐formed ring‐like structure in HeLa (Figure 1B), vs. 55% in SiHa (Figure S1B), vs. 52% in CaSki (Figure S1D). In contrast, 14% of nucleoli was surrounded by H3K9me3‐formed ring‐like structure in HeLa (Figure 1B) vs. 10% in SiHa (Figure S1B) vs. 19% in CaSki (Figure S1D). Together, in contrast to H3K9me3‐enriched heterochromatin, H3K27me3 surrounds the nucleolus to form an approximately closed circular ring. This unique spatial organization suggests that H3K27me3‐enriched PNH formation and nucleolus exert mutual functional effects on each other.

H3K27me3 aggregates encircling the nucleolus prompted us to study the relationship between PRC2 complexes and the nucleolus. Firstly, as a core component of PRC2.1 and PRC2.2, EZH2 aggregated in the peri‐nucleolar region and partially colocalized with NPM1 in HeLa (Figure 1C, middle), SiHa (Figure S2A), and CaSki (Figure S2B), similar to the distribution pattern of H3K27me3 (Figure 1A,B). Besides, as PRC2.1 subcomplex regulatory factors, PCL2 accumulated as a ring‐like structure in the peri‐nucleolar region (Figure 1C, upper), but neither PCL1 nor PCL3 presented ring‐like localization in this region predominantly (data not shown). However, the PRC2.2 subcomplex regulatory factor AEBP2 mainly localized at the nuclear periphery without colocalization with NPM1 (Figure 1C, bottom).

The co‐immunoprecipitation (co‐IP) assay showed that PCL2 interacted with EZH2 and NPM1 (Figure 1D,E). In contrast, AEBP2 did not interact with NPM1 (Figure 1D, Figure S2C–F). Besides, high‐resolution imaging of the nucleolus in HeLa cells further showed that exogenous EGFP‐PCL2 partially colocalized with mCherry‐NPM1 (Figure 1F), and the mCherry‐NPM1‐marked nucleolus was encircled by EGFP‐PCL2, consistent with the subcellular localization of endogenous PCL2 and NPM1 (Figure 1C). These results indicated that H3K27me3 aggregates encircling the nucleolus were mediated by the PRC2.1(PCL2) subcomplex, which also connects H3K27me3‐enriched heterochromatin and the nucleolus.

### 2.2 PCL2 Undergoes Liquid‐Liquid Phase Separation Via Predicted IDR (pIDR)

**FIGURE 2:**
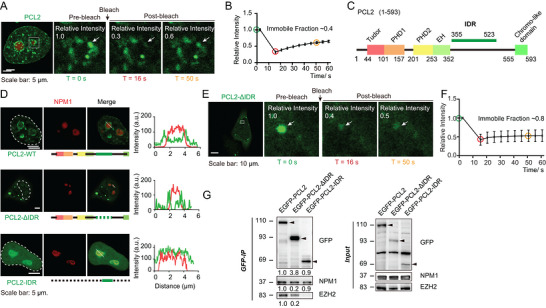
IDR is responsible for phase separation of PCL2 and regulates PCL2 interacting with other proteins. The dynamic intensity of EGFP‐PCL2 aggregates was analyzed using the FRAP assay in HeLa cells. The nucleolar boundary was indicated by mCherry‐NPM1. The areas containing EGFP‐PCL2 aggregates in the nucleolar periphery are marked by white rectangles. EGFP‐PCL2 foci (marked with a white arrow) were photobleached using 35% laser power for 4 cycles. Representative images of EGFP‐PCL2 aggregates within the white rectangle before and after photobleaching in live cells are shown. (B) Recovery kinetics of EGFP‐PCL2 aggregates from (A) are shown. Data are derived from three FRAP events, and the values are the mean ± SD. (C) Schematic diagram showing multiple domains in PCL2. The rectangle shapes with different colours indicated various domains: Tudor (magenta), plant homeodomain 1 (PHD1, orange), plant homeodomain (PHD2, yellow), winged‐helix domain (EH, grass green), and Chromo‐like domain (bright green). Green solid lines indicate IDR. (D) Left panels: Subcellular localization of EGFP‐PCL2‐WT/ΔIDR/IDR was shown in the left panels. The dashed line (green or black) indicates deleted amino acid fragments in the truncated PCL2. The nucleolus was labelled with mCherry‐NPM1. The dashed white lines mark the boundaries of the nuclear. Right panels: Intensity profiles of white lines across the nucleolus in the corresponding images (left panel) are shown. (E, F) The dynamic intensity of EGFP‐PCL2‐ΔIDR aggregates was analyzed using the FRAP assay in HeLa cells. EGFP‐PCL2‐ΔIDR aggregates in the cytoplasm (E) were analyzed and plotted (F) according to the methods in (A, B). Data are from three FRAP events, and the values are mean ± SD. (G) Co‐IP was performed in HeLa cells. Cells were transfected with plasmids expressing EGFP‐tagged PCL2‐WT/ΔIDR/IDR, and cell lysates were immunoprecipitated using GFP‐magnetic beads. Immunoblotting was performed to detect EGFP‐tagged protein, endogenous EZH2, and NPM1.

The nucleolus undergoes LLPS, which is crucial for its functions and is regulated by nucleolar proteins, heterochromatin‐associated proteins, noncoding RNAs, and intracellular salt concentrations [1, 21, 34, 35, 36]. Multivalent interactions between nucleic acids and nucleolar proteins, including FBL, NPM1, and URB1, established the multiphase liquid condensate in the nucleolus [10, 13, 37]. Furthermore, HP1, an H3K9me3 reader, aggregates in the nucleolar periphery via phase separation to seclude pericentromeric constitutive heterochromatin and regulate the integrity of the nucleolus [21, 38]. As a regulatory factor of H3K27me3, PCL2 accumulates around the nucleolus to form aggregates (Figure 1C), which were identified as liquid‐like condensates in vivo. Through FRAP assay in living cells, the fluorescence intensity of PCL2 condensates decreased to ∼30% after photobleaching, and recovered to ∼60% (Figure 2A,B).

Through protein expression and purification in vitro, EGFP‐PCL2 formed droplets at various KCl concentrations, with 10% crowding agent PEG‐8000. EGFP‐PCL2 formed droplets prominently in the buffer with 150 mM KCl, but the size of EGFP‐PCL2 droplets in 75 or 300 mM KCl is smaller (Figure S3A). Under the fixed condition of 150 mM KCl and 10% PEG‐8000, EGFP‐PCL2 droplets formed in a concentration‐dependent manner (Figure S3B). Moreover, the droplets exhibited gel‐like characteristics in vitro through the FRAP assay, which is difficult to recover (Figure S3C), compared with PCL2 condensates in living cells (Figure 2A,B).

To identify the critical domain of PCL2 responsible for phase separation, we used the protein disorder predictor IUPred2A to predict the intrinsically disordered region (IDR) [39]. An IDR spanning amino acid (aa) residues 355–523 is discovered (Figure 2C, Figure S3D). PCL2 mutants were generated, including PCL2‐ΔIDR (IDR deletion) and PCL2‐IDR (only retains IDR) (Figure 2D). Live‐cell imaging showed that PCL2‐ΔIDR was in the nucleus and cytoplasm, with partial colocalization with mCherry‐NPM1 (Figure 2D, middle). PCL2‐IDR mainly accumulated in the nucleolus, colocalized with mCherry‐NPM1 (Figure 2D, bottom). Additionally, the FRAP assay was applied to detect whether PCL2‐ΔIDR aggregates undergo phase separation (Figure 2E,F), while ∼ 80% of PCL2‐ΔIDR condensates were immobile, compared to ∼ 40% of PCL2‐WT (Figure 2A,B), indicating the important role of IDR in regulating the phase separation of PCL2. Colocalization between mCherry‐NPM1 and EGFP‐PCL2‐IDR prompted us to identify the domain in PCL2 responsible for PCL2‐NPM1 interaction. Co‐IP assay revealed that PCL2‐ΔIDR remained capable of interacting with EZH2 and NPM1, indicating that the IDR is not strictly essential for PCL2 binding to EZH2 and NPM1. In addition, the interaction strength between PCL2‐IDR and NPM1 (ratio = 0.9) was comparable to that of PCL2‐WT (ratio = 1), but PCL2‐IDR does not interact with EZH2 (Figure 2G). Together, we identified the critical role of IDR in PCL2 aggregation in the peri‐nucleolar region with a phase‐separation manner, and PCL2 interacting with the core component of PRC2 (EZH2) as well as nucleolar factor (NPM1). The result suggested that IDR in PCL2 is essential for phase separation of PCL2, but insufficient to encircle the nucleolus by forming abundant liquid‐like condensate like PCL2‐WT.

### 2.3 PRC2.1 Maintains Nucleolar Morphology Via Facilating H3K27me3‐Enriched PNH Formation and NPM1 Pentamerization

**FIGURE 3:**
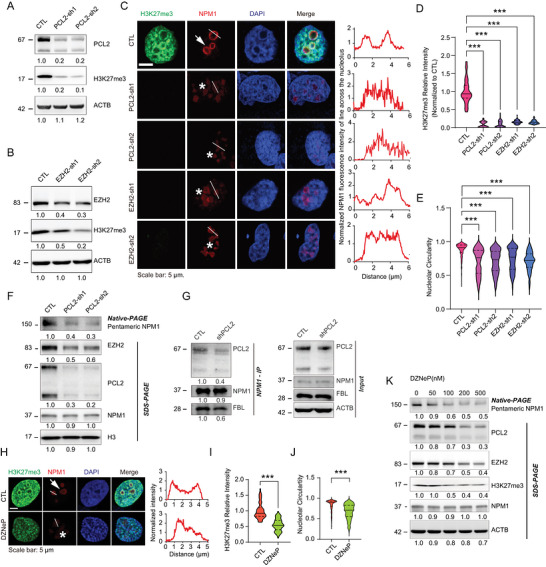
H3K27me3 and pentameric NPM1 were decreased by PRC2.1 deficiency or PRC2 inhibitor, with destroyed nucleolar structure. (A, B) PCL2‐, EZH2‐KD was conducted via lenti‐virus containing specific shRNA, including PCL2‐sh1/PCL2‐sh2 targeting PCL2 and EZH2‐sh1/EZH2‐sh2 targeting EZH2, respectively. Empty vector pLKO.1 was used as a negative control (CTL). The levels of PCL2, EZH2, and H3K27me3 proteins were analyzed by immunoblotting and normalized to β‐actin (ACTB). The relative protein levels were indicated under the bands, with the CTL group as 1. (C) Left panel, subcellular localization of H3K27me3 (green), NPM1 (red), and DAPI (blue) in HeLa cells is shown in the representative IF images, following PCL2 or EZH2 knockdown. Relative fluorescence intensity profiles of NPM1 along the indicated white lines across the nucleolus are shown in the right panels. The *Y*‐axis represents fluorescence intensity (a.u.) of each point along the line, which was normalized to the average value of the total points, and the *X*‐axis represents the distance along the line (µm). (D) Relative fluorescence intensity of H3K27me3 in PCL2‐KD and EZH2‐KD cells from (C) was quantified and normalized to the CTL group. Cells in each group (n > 60) were collected from three independent biological experiments for statistical analysis. Significance was assessed using the Kruskal‐Wallis test followed by Dunn's multiple comparisons test. (E) Nucleolar circularity in (C) was quantified, and the total number of nucleoli in each group is greater than 200. Data were collected from three independent biological experiments. Statistical significance was determined using the Kruskal‐Wallis test followed by Dunn's multiple comparisons test. (F) Pentameric NPM1 in PCL2‐KD and control cells (CTL) was analysed by Native‐PAGE and immunoblotting. The levels of PCL2, EZH2, and NPM1 proteins were analyzed by immunoblotting and normalized to β‐actin (ACTB). (G) HeLa cells were transfected with the shPCL2 pool (PCL2‐sh1 and PCL2‐sh2), with co‐IP assay performed to detect the interaction among NPM1, FBL, and PCL2. Cell lysates were immunoprecipitated with a mouse anti‐NPM1 antibody, and the intensity of NPM1, FBL, and PCL2 in the IP product was detected and normalized. (H) Right panels: H3K27me3 (green), NPM1 (red), DAPI (blue) staining in DZNeP (100 nM)‐/DMSO‐treated cells. Left panels: The rim enrichment of NPM1 from the representative was shown. (I) Relative fluorescence intensity of H3K27me3 in DZNeP‐treated cells was quantified and normalized to CTL. Cells in each group (n > 60) were collected from three independent biological experiments. Statistical significance was assessed using the Mann‐Whitney U test. (J) Nucleolar circularity in each group, shown in (H), was quantified. The total number of nucleoli in each group is greater than 200. Data were collected from three independent biological experiments. Statistical significance was determined using the Mann‐Whitney U test. (K) The relative level of PCL2, EZH2, H3K27me3, and total NPM1 was detected by SDS‐PAGE, and the NPM1 pentamer was detected by Native‐PAGE. The relative level of proteins is indicated under the bands, with the CTL group as 1. White arrows and stars denote regular and irregular NPM1‐marked nucleolus, respectively. ^***^, *P* < 0.001.

In the nucleolar periphery, H3K27me3, accompanied by its catalytic complex PRC2.1, accumulated as a ring‐like structure (Figures 1 and 2). Therefore, the total level and subcellular localization of H3K27me3 were analyzed following PRC2.1 subunit knockdown (KD). Compared with the control group, PCL2‐shRNA and EZH2‐shRNA decreased PCL2 and EZH2 expression levels, respectively (Figure 3A,B). Either PCL2‐ or EZH2‐shRNA decreased the level of H3K27me3 to 20–50%, normalized to control (Figure 3A,B), and the fluorescence intensity of H3K27me3 in the nucleus were decreased significantly (*P* < 0.001) (Figure 3C,D). The peri‐nucleolar ring‐like structure formed by H3K27me3 was almost invisible (Figure 3C), verifying that both PCL2 and EZH2 were essential for modification, aggregation, and subcellular localization of H3K27me3. Unexpectedly, the pattern of NPM1 aggregates in the nucleus was irregular in both PCL2‐KD and EZH2‐KD cells (Figure 3C,E). In the control group (CTL), the “M‐shaped” profile reflects the characteristic rim enrichment of NPM1 at the nucleolar periphery. In PCL2‐, EZH2‐KD cells, the profiles appear flattened or fragmented, and the nucleolus presents irregular characteristics compared to CTL, as reflected by reduced circularity compared with the CTL (Figure 3C,E). The critical role of PRC2.1 subcomplex in mediating H3K27me3 modification and regulating subcellular localization was further verified in CaSki and SiHa cells (Figure S4A–F). Interestingly, compared with the negative shRNA control (CTL), the expression level (Figure 3F), as well as the fluorescence intensity of EZH2 (Figure S4G,H), was decreased significantly in PCL2‐deficient cells (*P* < 0.001), with fewer aggregate formations.

Furthermore, the irregular NPM1‐labelled nucleolus prompted us to examine pentameric NPM1, the key functional unit maintaining the nucleolar structure. Immunoblotting assay showed that the level of pentameric NPM1 was decreased in both PCL2‐KD and EZH2‐KD cells (Figures 3F, S4I–M), compared with the control cells. To further investigate the impact of PCL2 on interactions between NPM1 and Fibrillarin (FBL), we performed co‐IP assay with PCL2‐KD cells and control cells. The results showed that both NPM1‐FBL and NPM1‐PCL2 interaction level in PCL2‐KD cells was decreased, compared with the control cells (Figure 3G; and Figure S4N,O). The morphological alteration was accompanied by reduced NPM1 pentamer and diminished interaction between NPM1 and FBL, indicating that PRC2.1(PCL2) deficiency destroyed nucleolar integrity. To verify the essential role of PRC2.1 in H3K27me3 modification and NPM1 pentamerization, 3‐deazaneplanocin A (DZNeP, an inhibitor of EZH2) was applied in HeLa, SiHa, and CaSki cells. Immunoblotting assay showed that protein levels of EZH2, PCL2, H3K27me3, and pentameric NPM1 were decreased by DZNeP treatment (Figure 3H–J) in a dose‐dependent manner (Figures 3K, S4P,Q), while NPM1 total level remained unchanged (Figure 3K). Consistent with the PCL2‐KD and EZH2‐KD results, DZNeP (100 nM) treatment resulted in reduced fluorescence intensity of H3K27me3 in the nucleus (Figure 3H,I, *P* < 0.001), with an invisible peri‐nucleolar structure of H3K27me3 (Figure 3H), as well as an irregular NPM1‐marked nucleolus (Figure 3J).

**FIGURE 4:**
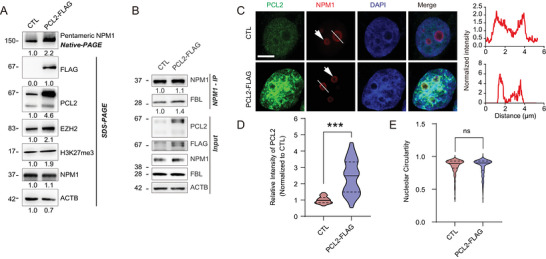
H3K27me3 and NPM1 pentamerization are enhanced by PCL2 overexpression. (A) PCL2 overexpression was performed using lentivirus in the PCL2‐FLAG HeLa cells. Upper: Following Native‐PAGE and immunoblotting, the level of pentameric NPM1 in the PCL2‐FLAG group was measured and normalised to ACTB. Bottom: Following SDS‐PAGE, the protein levels of FLAG, PCL2, EZH2, NPM1, and H3K27me3 were detected by immunoblotting. Relative protein levels are indicated under the bands. (B) Co‐IP assay in PCL2‐overexpressed HeLa cells. Cells were lysed and immunoprecipitated with a mouse anti‐NPM1 antibody. Immunoblotting was performed to detect interaction between NPM1 and FBL. (C) Right panels: PCL2 (green), NPM1 (red), and DAPI (blue) staining in PCL2‐FLAG and CTL groups. Representative images are shown. White arrows indicated regular NPM1‐marked nucleoli. Left panels: The rim enrichment of NPM1 from the representative was shown. (D) Relative fluorescence intensity of PCL2 in PCL2‐FLAG and CTL cells from (C) was quantified and normalized to the CTL group, shown in (C) (n > 60). Data were collected from three independent biological experiments. Statistical significance was determined using the Mann‐Whitney U test. (E) Nucleolar circularity in each group, shown in (C) (nucleoli number > 200), was quantified. Data were collected from three independent biological experiments. Statistical significance was determined using the Mann‐Whitney U test. ns, not significant, ns, not significant, ^***^, *P* < 0.001.

Notably, compared with control cells, not only EZH2 and H3K27me3 levels, but also NPM1 pentamer, were up‐regulated in PCL2‐overexpressing cells (Figure 4A, Figure S5A–D), in which stronger interaction between NPM1 and FBL was also detected (Figure 4B, Figure S5E,F). The fluorescence intensity of PCL2 in the nucleus was increased by lentivirus‐mediated PCL2 overexpression (PCL2‐FLAG), compared with the empty vector control (CTL) (Figure 4C,D, Figure S5A,B). However, exogenous PCL2‐FLAG did not alter the morphology of NPM1‐marked nucleoli, as shown in nucleolar rim enrichment (Figure 4C, right) and nucleolar circularity analysis (Figure 4C,E). Furthermore, the expression level and localization of EZH2 are correlated with PCL2 (Figure 3, Figure S4), which prompted us to analyze the compensatory effect of PCL2 overexpression on EZH2 deficiency. Consistent with the disruption of endogenous NPM1‐labelled nucleoli by EZH2‐shRNA (Figure 3C), mCherry‐NPM1‐labelled nucleoli present a fragmented state (Figure S5G, upper), in which nucleolar circularity decreased compared to CTL (Figure S5H), and exogenous expression of PCL2 partially attenuates this phenomenon (Figure S5G, bottom, Figure S5H), indicating that overexpression of PCL2 attenuated the disruption effect of EZH2 deficiency on the nucleolar morphology. Altogether, the PRC2.1 subcomplex plays a critical role in NPM1 pentamerization, as well as NPM1‐labelled nucleolar morphology maintenance.

### 2.4 PRC2.1 Enhances rRNA Synthesis, Global Protein Expression, and Cell Cycle Progression

**FIGURE 5:**
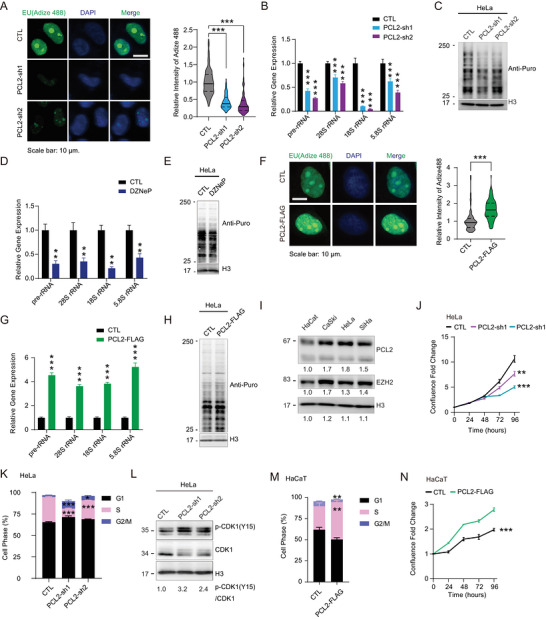
PCL2 is essential for rRNA and protein synthesis, as well as cell proliferation. (A) EU‐labelled newly synthesised RNAs were clicked and captured in shRNA‐mediated PCL2‐KD cells. Representative images are shown in the left panel, with relative intensity shown in the right panel. Cells in each group (n > 60) were collected from three independent biological experiments for statistical analysis. Significance was assessed using the Kruskal‐Wallis test followed by Dunn's multiple comparisons test. (B, C) HeLa cells were transfected with shRNA targeting PCL2, with an empty vector serving as the negative control (CTL). Relative expression levels of pre‐ and mature rRNAs (28S/18S/5.8S), normalised to GAPDH, were measured by qRT‐PCR. Data presented are means ± SD from three biological replicates. Data are presented as mean ± SD. Statistical significance was analyzed by one‐way ANOVA followed by Dunnett's post hoc test (B). Nascent peptides were labelled with puromycin and detected using an anti‐puromycin antibody. The global protein synthesis level of PCL2‐KD HeLa cells was measured. Histone H3 was used as a reference (C). (D, E) HeLa cells were treated with 100 nM DZNeP, with DMSO as a control. qRT‐PCR was performed to measure the levels of pre‐ and mature rRNAs. Statistical significance was determined using an unpaired two‐tailed Student's *t*‐test (D). Immunoblotting was conducted to evaluate the levels of global protein synthesis (E). (F‐H) PCL2‐FLAG was transfected into HeLa cells, with an empty vector as a control. Representative images in the left panel of newly synthesised RNA; relative intensity in the right panel and statistical significance was determined using the Mann‐Whitney U test (cells in each group > 60) (F); rRNA level with statistical significance was determined using an unpaired two‐tailed Student's *t*‐test (G), and global protein synthesis (H) was detected by EU, qRT‐PCR, and immunoblotting, respectively. (I) The relative levels of PCL2 and EZH2 in non‐cancer cells/epidermal keratinocyte line (HaCaT) or cervical cancer cells (CaSki, HeLa, SiHa) were detected by immunoblotting, with histone H3 as a reference control. (J) HeLa cells were transfected with shRNA to knock down PCL2. At 0/24/48/72/96 h post‐transfection (hpt), cell proliferation of PCL2‐KD and CTL groups was analysed by CCK8 assay. The *y*‐axis represents the OD450 value relative to the confluence at 0 hpt. Error bars represent mean ± SD from three biological replicates. Statistical significance was determined by one‐way ANOVA followed by Dunnett's post hoc test. (K, L) HeLa cells were transfected with shRNA to knock down PCL2. At 48 hpt, EdU‐labelled newly synthesised DNA was stained by Adize 555, and total DNA was stained by DAPI, followed by flow cytometry. The percentage of cells at G2/M in each group was analysed and normalised to the CTL group. Data are presented as mean ± SD. Statistical significance was determined by one‐way ANOVA followed by Dunnett's post hoc test. (K). The levels of p‐CDK1(Y15) and CDK1 were detected with the corresponding antibodies and normalised to histone H3. The relative ratio of p‐CDK1(Y15)/CDK1 was calculated and noted under the bands (L). (M, N) HaCaT cells were transfected with PCL2‐FLAG to overexpress PCL2. At 48 hpt, cells were collected and analysed by flow cytometry (M). At 0/24/48/72/96 h post‐transfection (hpt), cell proliferation of PCL2‐FLAG and CTL groups was tested with CCK8 assay. Statistical significance was analyzed using an unpaired two‐tailed Student's *t*‐test (N). ^*^, *P* < 0.05; ^**^, *P* <0.01; ^***^, *P* < 0.001.

The nucleolus undergoes dynamic assembly and disassembly throughout the cell cycle, resulting in diverse nucleolar morphology affecting rRNA transcription activity. Our finding that PCL2 in the PRC2.1 subcomplex plays an essential role in H3K27me3‐marked PNH establishment and nucleolar integrity suggests that PCL2 is involved in nucleolus‐associated biological processes. Moreover, PCL2 is also critical for NPM1 pentamerization and interaction between NPM1 and FBL (Figures 3 and 4), which maintains the periphery of the nucleolar GC region and regulates nucleolar functions [11, 40]. Therefore, the critical role of PRC2.1(PCL2) in rRNA biogenesis was investigated in the following experiments. Compared with the control (CTL) group, EU‐labelled newly synthesised RNA was significantly decreased in PCL2‐KD cells (Figure 5A). Consistently, the rRNA expression level, including pre‐rRNA and mature rRNA (28S, 18S, 5.8S) (Figure 5B), as well as global protein synthesis (Figure 5C), was significantly decreased in PCL2‐KD compared with the control (CTL) group. Similar reductions in rRNA levels and *de novo* protein synthesis were observed following PCL2 knockdown in CaSki and SiHa cells (Figure S6A–J). Consistent with PCL2 knockdown, DZNeP treatment diminished the levels of rRNA and *de novo* protein significantly in HeLa cells (Figure 5D,E), CaSki and SiHa cells (Figure S6D,E,I,J). Conversely, synthesis of rRNA and *de novo* protein was enhanced by PCL2 overexpression in HeLa (Figure 5F–H), CaSki and SiHa cells (Figure S6K–N).

Remarkably, compared with non‐cancer cells (HaCaT), higher expression levels of PCL2 and EZH2 were detected in cervical cancer cells (CaSki, HeLa, SiHa) (Figure 5I). In addition, the Timer2.0 database was used to analyse the relative mRNA levels of PCL2 in the CESC patients. Indeed, the transcription activity of PCL2 was significantly higher in cancer tissues vs. para‐carcinoma tissues (*P* < 0.05) (Figure S7A), indicating that PCL2 is associated with high proliferation of cancer cells. Colony formation (Figure S7B) and CCK‐8 assay (Figure 5J), respectively, showed that PCL2 knockdown decreased cell proliferation significantly (*P* < 0.01). Additionally, flow cytometry was conducted, and the results showed that PCL2 knockdown caused G2/M phase arrest (Figure 5K, Figure S7C,D). Phosphorylated (p)‐CDK1 (pY15), as a negative regulator of G2/M transition [41], was upregulated in PCL2‐KD cells (Figure 5L, Figure S7C,D). In PCL2‐KD cells, we also detected decreased mRNA levels of *AURKA* and *PLK1* (Figure S7E), which are positive regulators of cell cycle transition into the mitotic phase [41, 42]. Moreover, the percentage of apoptotic cells was increased in PCL2‐KD cells, compared with control cells (Figure S7F–H). Conversely, in HaCaT cells, which have a lower endogenous PCL2 expression level, exogenous PCL2 significantly decreased the percentage of cells in G1 and G2/M phase (Figure 5M, *P* < 0.01) but significantly increased the fold change in cell confluence (Figure 5N, *P* < 0.001), indicating that exogenous PCL2 promotes proliferation in non‐cancer cells. Collectively, PCL2 plays a necessary role in rRNA synthesis, a key nucleolar biological process that is critical for global protein synthesis. In addition, PCL2 promotes hyper‐proliferation of cervical cancer cells, suggesting that PCL2 is a potential diagnostic indicator and therapeutic target of tumorigenesis.

### 2.5 PCL2 Contributes to NPM1 Pentamerization in an IDR‐Dependent Manner

**FIGURE 6:**
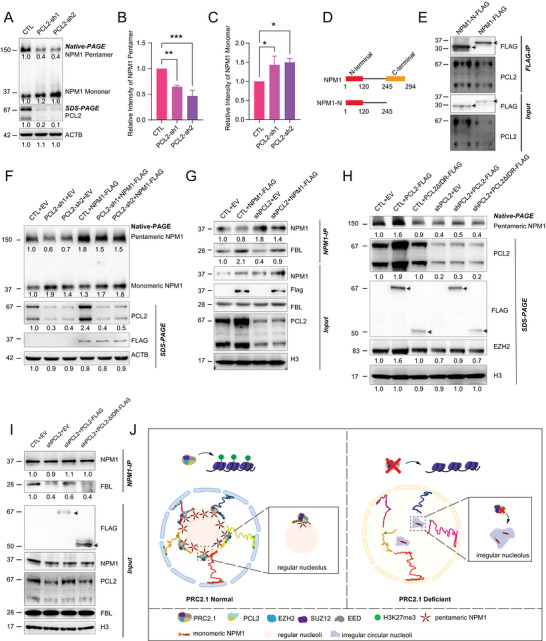
PCL2 regulates NPM1 pentamerization in an IDR‐dependent manner.(A) The relative level of PCL2 was detected by SDS‐PAGE, while the relative level of NPM1 pentamer and NPM1 monomer was detected by Native‐PAGE. ACTB is loading control. The relative level of proteins is indicated under the bands, with CTL group as 1. (B, C) Relative expression level of pentameric NPM1 (B) and monomeric NPM1 (C) were measured by ImageJ and analyzed by ANOVA in GraphPad. The value is mean ± SD from three independent biological experiments. (D) Schematic representation of full‐length NPM1 and its truncation constructs used in this study. (E) Co‐IP was performed in HeLa cells. Cells were transfected with plasmids expressing NPM1‐FLAG and NPM1‐N‐FLAG, and cell lysates were immunoprecipitated using FLAG‐magnetic beads. Immunoblotting was performed to detect the FLAG‐tagged protein, endogenous PCL2. (F, G) SiHa cells were transfected with shRNA targeting PCL2, with the negative shRNA as control. At 12 hpt, the cells were subsequently transfected with exogenous NPM1‐FLAG or an empty vector. At 48 hpt, the levels of pentameric NPM1, monomeric NPM1, PCL2, and FLAG were detected with the indicated antibodies, with ACTB as a loading control (F). In parallel assay, cell lysates were immunoprecipitated with a mouse anti‐NPM1 antibody. Immunoblotting was performed to detect interaction between NPM1 and FBL at 48 hpt (G). (H, I) SiHa cells were transfected with shRNA targeting PCL2, with the negative shRNA as control. At 12 hpt, the cells were subsequently transfected with exogenous PCL2‐FLAG or PCL2‐ΔIDR‐FLAG. At 48 hpt, Native‐PAGE, SDS‐PAGE as well as co‐IP were performed as described in (F, G). (J) A working model illustrating the mechanism of PRC2.1(PCL2) regulating the nucleolus. ^*^, *P* < 0.05; ^**^, *P* < 0.01; ^***^, *P* < 0.001.

The data above suggested PCL2 plays critical roles in maintaining nucleolar morphology via peri‐nucleolar heterochromatin organization and NPM1 pentamerization (Figures 3 and 4). Moreover, SDS‐PAGE data showed that PCL2‐KD does not affect the total level of NPM1 (Figure 3 and 4, Figure S8A,B), but Native‐PAGE analyses revealed that PCL2‐KD resulted in a reduction of pentameric NPM1, accompanied by monomeric NPM1 increasing at 48 hpt (Figure 6A–C), indicating that PCL2 primarily regulates NPM1 assembly rather than its expression.

Besides, time‐course analysis of pentameric NPM1 further showed that a reduction in NPM1 pentamerization occurs prior to a decrease of H3K27me3 and rRNA synthesis level (Figure S8C–E), suggesting that these events are not the initial drivers of NPM1 disassembly. To further identify the function of PRC2 in nucleolar integrity maintenance via an H3K27me3‐dependent manner or a PCL2‐dependent manner. Selective PRC2 inhibitors EPZ‐6438 and EED226, which inhibit catalytic activity without significantly degrading the complex [43, 44], were applied to detect the potential mechanism. We compared PCL2 deficiency and selective PRC2 inhibitors treatment at matched early time point. As shown above, treatment with EPZ‐6438 or EED226 for 24 h effectively reduced H3K27me3 levels. However, under these conditions, PCL2, EZH2 and NPM1 pentamer remained unreduced (Figure S8F). In contrast, PCL2 knockdown at the same time point (24 h) led to a reduction in pentameric NPM1, despite no detectable decrease in H3K27me3 levels (Figure S8C,F). These findings indicate that the reduction of H3K27me3 alone is not sufficient to drive the early disruption of NPM1 pentamerization. Consistently, prolonged PRC2 inhibition (48 h) resulted in a moderate decrease in pentameric NPM1 (Figure S8G) and nucleolar circularity (Figure S8H), suggesting that H3K27me3 may contribute to nucleolar organization at later stage, potentially through indirect effects on chromatin architecture. These findings indicate that PCL2 regulates NPM1 pentamerization at early stages, while H3K27me3 contributes in a delayed and cooperative manner to maintain nucleolar integrity.

To explore the molecular mechanism of PCL2 regulating NPM1 pentamerization, we further generated an NPM1 truncated construction (NPM1‐N) containing the N‐terminal domain (Figure 6D), which is responsible for NPM1 pentamerization [45]. Co‐IP assay showed that NPM1‐N interacts with PCL2 (Figure 6E), indicating that PCL2 stabilizes NPM1 pentamerization. To further assess the functional relevance of NPM1‐PCL2 interaction, we performed rescue experiments by overexpressing NPM1 in PCL2 knockdown cells. Notably, ectopic expression of NPM1 partially restored pentameric NPM1 levels (Figure 6F, Figure S8I) and the interaction between NPM1 and the nucleolar protein FBL (Figure 6G), supporting a role for PCL2 in stabilizing nucleolar integrity. The expression level of PCL2 was upregulated by exogenous NPM1‐FLAG (Figure S8K), compared with the empty vector, but reduced upon NPM1‐targeting shRNA knockdown (Figure S8J), showing that NPM1 has a positive role in the expression of PRC2.1 subunits, indicating a bidirectional regulatory relationship between PCL2 and NPM1.

Given that the IDR of PCL2 is critical for its phase separation properties and interactions with PRC2 components and NPM1 (Figure 2), we further examined whether the IDR is required for NPM1 pentamerization. Similar to the EGFP‐tagged PCL2 mutant, the PCL2‐ΔIDR‐FLAG retain weaken interaction with EZH2 and NPM1 (Figure 3G, Figure S8L). Substantially, in PCL2‐KD cells, exogenous overexpression of PCL2‐FLAG, but not the PCL2‐ΔIDR‐FLAG, partially restored the pentameric NPM1 levels and NPM1‐FBL interaction (Figure 6H,I), demonstrating that IDR is essential for PCL2 to stabilize nucleolar integrity.

Based on these findings, we propose a working model for PRC2.1(PCL2) to regulate the nucleolus. PRC2.1(PCL2) localizes to the peri‐nucleolar region, forming liquid‐like condensates dependent on IDR, which is a critical region interacting with NPM1 and regulating NPM1 pentamerization. With sufficient PRC2.1(PCL2), enough NPM1 pentamers and H3K27me3 can preserve nucleolar structure. Conversely, a lack of PRC2.1(PCL2) may lead to a decrease in H3K27me3, failure of NPM1 pentamer formation, and irregular nucleolus shapes (Figure 6J).

## 3 Discussion

The nucleolus plays multiple roles in cellular functions, including cell cycle regulation, ribosome biogenesis, and cell differentiation. Numerous studies have shown that nucleolar morphology and function were determined by nucleolar proteins (NPM1, FBL, and URB1), ribosomal RNA, heterochromatin and its associated proteins [21, 35, 36], and long non‐coding RNAs (LETN, SLERT) [7, 11, 37, 46, 47, 48]. As is known, both heterochromatin and nucleolus are located in the nucleus, and some studies suggest a mutual effect between heterochromatin and nucleolar proteins [21, 35, 36, 49, 50]. However, the detailed regulatory mechanism of H3K27me3‐enriched PNH on the physiological activity of the nucleolus remains incompletely understood.

In this study, we found that H3K27me3‐enriched PNH, together with its catalytic complex PRC2, accumulated around the nucleolus to form an approximate closed ring structure (Figures 1, 2, 3, 4). This distribution suggests a close spatial relationship between H3K27me3‐enriched PNH and the nucleolus, rather than direct suppression of rDNA transcription by H3K27me3. These observations prompted us to investigate the regulatory role of PRC2 on H3K27me3‐enriched PNH formation and nucleolar activity. We identified PCL2, an accessory subunit of the PRC2.1 subcomplex, as a key regulatory factor enriched at the nucleolar periphery. PCL2 interacts with the nucleolar protein NPM1 and localizes to regions adjacent to the nucleolus (Figures 2, 4, and 5).

Impaired rRNA processing is known to alter the nucleolar morphology [51]. In this manuscript, deficiency of PRC2.1(PCL2) disrupts nucleolar morphology and reduces NPM1 pentamerization (Figure 3), accompanied by reduced rRNA synthesis as well as global protein synthesis (Figure 5). Importantly, these phenotypes were further validated in primary cells (Figure S9), where PCL2 deficiency similarly led to impaired NPM1 pentamerization (Figure S9A,H), nucleolar disruption (Figure S9B–D) and dysfunction of the nucleolus (Figure S9G,I). These findings suggest that the role of PRC2.1(PCL2) in regulating nucleolar functions is not restricted to transformed cell lines, but may represent a conserved mechanism underlying nucleolar homeostasis. While these changes occur without significant alteration in total NPM1 abundance (Figure S8A,B), indicating that PCL2 primarily affects through modulation of protein assembly and spatial organization rather than protein expression levels.

### 3.1 PCL2 Condensates and Peri‐Nucleolar Chromatin Organization

Proteins and/or nucleic acids aggregate or segregate dynamically to form droplet‐like condensates [52], which are commonly observed in eukaryotic cells and regulate diverse cellular processes, including membrane‐less organelle organization [1], (hetero)chromatin formation [53], transcriptional regulation [54, 55], and signal recognition [2]. LLPS could also drive heterochromatin compartmentalization, such as constitutive heterochromatin foci at centromeres mediated by HP1 [56, 57], 53BP1 [58], methyl‐CpG binding protein 2 (MeCP2) [59], scaffold attachment factor B (SAFB) [60], constitutive heterochromatin at telomeres regulated by TRF1/2 [61], and facultative heterochromatin in X chromosome inactivation induced by RNA‐binding proteins [62]. Facultative heterochromatin plays a critical role in regulating the accessibility of DNA, associating with cell division and differentiation [63, 64, 65]. In this study, we found that PCL2, a heterochromatin‐associated protein, undergoes phase separation via IDR, which also enables PCL2 to interact with NPM1 and other components of PRC2.1. Our findings suggest that PCL2‐mediated condensation is likely to contribute to the spatial confinement and molecular organization of PRC2.1 complex, but also H3K27me3‐enriched chromatin in nucleolar periphery (Figures 1, 2, 3, 4). Furthermore, our data revealed that PCL2 has a regulatory effect on the expression of chromatin remodeling factors and chromatin positioning (data not shown). Moreover, the potential role of other Polycomb‐like (PCL) family members in nucleolar functions warrants further consideration. Our correlation analysis between PCLs and nucleolar protein‐coding genes revealed that PCL3 exhibits a positive relationship with several key nucleolar components (Figure S10A,B). This observation suggests that PCL3 may also participate in maintaining nucleolar homeostasis under specific physiological or pathological contexts. Given the structural homology and functional diversity among PCL family members, whether PCL3 modulates nucleolar dynamics through similar phase separation mechanisms or distinct protein interaction networks remains an intriguing question for future research.

### 3.2 PRC2.1(PCL2) Maintains the Morphology and Biological Function of the Nucleolus Via H3K27me3‐Enriched PNH and NPM1 Pentamerization

Heterochromatin, as well as heterochromatin‐associated proteins (such as HP1, Lamin), have been implicated in nucleolar condensate formation and the physiological activity of the nucleolus [21, 35, 36, 50]. Our data extends this concept by implicating PRC2.1(PCL2) and H3K27me3‐enriched PNH as a novel chromatin environment and a flexible scaffold that contributes to nucleolar stability.

Notably, loss of PRC2.1 activity was performed via PCL2 knockdown and PRC2 inhibitor treatment (Figure 3, Figure S4), leading to disruption of nucleolar morphology, as well as reduction of NPM1 pentamerization and rRNA biogenesis. Time‐course analysis of pentameric NPM1 further indicated that reduced NPM1 pentamer can precede detectable defects in rRNA synthesis and H3K27me3, suggesting that perturbation of nucleolar protein assembly may represent an early event in nucleolar dysfunction (Figure 6, Figure S8). Hence, PRC2.1, H3K27me3‐enriched PNH, and nucleolar protein NPM1 form an intricate regulatory and interaction network, synergistically sustaining the structural and functional integrity of the nucleolus.

Furthermore, transcriptomic and metabolomic analyses reveal that PCL2 deficiency also affects nucleotide metabolism and cellular energy homeostasis, which is tightly coupled to nucleolar activity (data not shown). Together, these findings indicate that nucleolar dysfunction caused by PRC2.1 deficiency is a comprehensive and synergistic effect of multiple molecular events, including disordered chromatin organization, disrupted nucleolar protein assembly (eg, NPM1 oligomerization, NPM1‐FBL interaction), and metabolic constraints.

In summary, this study unveils that higher‐order nuclear architecture is established by two compositionally distinct condensates: heterochromatin and the nucleolus. We identify PRC2.1(PCL2) regulates nucleolar integrity through a dual mechanism: IDR‐dependent control of NPM1 pentamerization, and a secondary role in organizing peri‐nucleolar chromatin via PRC2.1‐mediated H3K27me3 deposition.

## 4 Methods

### 4.1 Antibodies for Western Blotting (WB), Immunofluorescence (IF), and Coimmunoprecipitation (co‐IP)

Antibodies used for this study were purchased from the indicated suppliers. The primary antibodies include anti‐NPM1 antibody (Proteintech, #60096‐1‐Ig; 1:400 dilution for IF, 1 µg for co‐IP, 1:10000 for WB), anti‐PCL2 antibody (Abcam, #ab254336; 1:100 dilution for IF, 1 µg for co‐IP, 1:2500 for WB), anti‐EZH2 antibody (Cell Signaling Technology, #D2C9; 1:100 dilution for IF, 1:1000 for WB), anti‐FBL antibody (Proteintech, #66985‐1‐Ig; 1:2500 for WB), anti‐AEBP2 antibody (BOSTER, #PB1029; 1:200 dilution for IF, 1:100 dilution for co‐IP, 1:5000 for WB), anti‐FLAG antibody (Proteintech, #66008‐4‐Ig; 1:10000 for WB), anti‐GFP antibody (Proteintech, #50430‐2‐AP, 1:10000 for WB), anti‐H3K27me3 antibody (Thermo Fisher, #MA5‐11198; 1:100 dilution for IF, 1:10000 for WB) and anti‐H3K9me3 antibody (Thermo Fisher, #49‐1008; 1:100 dilution for IF), anti‐Cyclin‐dependent kinase 1 (CDK1) antibody (Sigma–Aldrich, #ZRB1485; 1:2500 for WB), anti p‐CDK1(Tyr 15) antibody (Cell Signaling Technology, #10A11; 1:2500 for WB), anti‐puromycin antibody (Sigma–Aldrich, #MABE343; 1:2000 for WB). The secondary antibodies include goat anti‐mouse IgG (H+L) (Jackson ImmunoResearch, #115‐035‐003; 1:10000 dilution for WB), goat anti‐rabbit IgG (H+L) (Jackson ImmunoResearch, #111‐035‐003; 1:10000 dilution for WB), Alexa 488 goat anti‐rabbit (Jackson ImmunoResearch, #111‐545‐003; 1:300 dilution for IF), Alexa 568 goat anti‐mouse (Thermo Fisher, #A‐11004; 1:300 dilution for IF.

### 4.2 Plasmid Construction

The coding sequences for full‐length PCL2, NPM1, and truncated PCL2 were amplified individually from cDNAs isolated from HeLa cells, and inserted into the transient expression vector pcDNA3.1+/pEGFP‐C1/pmCherry‐C1/pCAG or the lentiviral vector pLVX for generating stable cell lines. The oligos containing specific shRNA sequences targeting PCL2, EZH2 and NPM1 were inserted into the pLKO.1‐TRC vector (Addgene). Primers for constructing plasmids are listed in Table S1.

### 4.3 Cell Culture, Transfection, and PRC2 Inhibitor Treatment

HEK‐293T and HeLa cells were cultured in DMEM with high glucose. CaSki cells were grown in RPMI 1640. SiHa cells were cultured in MEM supplemented with NEAA. All medium were supplemented with 10% FBS (Sigma–Aldrich), 1% penicillin, and 1% streptomycin (Solarbio). All cells were cultured in a humidified incubator at 37°C in 5% CO_2_. Expression plasmids were transfected into HeLa/CaSki/SiHa cells with jetPRIME (Polyplus) or transfected into HEK‐293T cells with PEI‐40K, according to the recommended methods. HeLa/CaSki/SiHa cells were treated with DZNeP (GLPBIO), EPZ‐6438 (MedChemExpress) and EED226 (MedChemExpress) at the indicated concentrations for 24 h or 48 h, while control cells were treated with DMSO.

### 4.4 Lentivirus Production and Construction of Stable Cell Lines

HEK‐293T cells were seeded onto 60‐mm plates 12 h before transfection. pMD2.G (1000 ng), pSPAX2 (2000 ng), together with the lentiviral vector (3000 ng, pLKO.1‐shPCL2/pLKO.1‐shEZH2/pLVX‐PCL2) were co‐transfected into HEK‐293T cells in each well using PEI‐40K, following the recommended protocol. At 48 h post‐transfection, the medium was harvested, filtered, and stored at 4°C or −80°C. HeLa/SiHa/CaSki cells were infected with the harvested lentivirus and treated with 1 µg/ml puromycin to obtain stable cell lines for PCL2/EZH2 knockdown or PCL2 overexpression.

### 4.5 Microscopy

Cells were seeded onto glass coverslips in a 12‐well plate and cultured overnight. The cells were transfected with the shRNA‐containing plasmid, infected with lentivirus, or treated with DZNeP, with the untreated cells used as a negative control. After 4% PFA fixation at room temperature (RT), cells were permeabilised with 0.5% TritonX‐100 and blocked with 3% BSA. Diluted mouse anti‐NPM1 antibody, rabbit anti‐PCL2 antibody, rabbit anti‐AEBP2 antibody, rabbit anti‐EZH2 antibody, or rabbit anti‐H3K27me3 antibody was applied to the coverslips and incubated overnight at 4°C. Corresponding fluorochrome‐conjugated secondary antibodies were used at room temperature for 1 h. The cells on glass coverslips were stained with DAPI and observed with a fluorescence microscope. Images were generally captured and analysed with NIS‐Elements AR Analysis (Nikon) or Zen Black (ZEISS), and high‐resolution images were captured with the Zeiss Elyra 7.

Cells were grown on glass‐bottom cell culture dishes (NEST) for live‐cell imaging and transiently transfected with EGFP‐PCL2‐WT/MT and/or mCherry‐NPM1. During imaging, the cells were cultured on a heated stage (37°C) and maintained in a humidified atmosphere containing 5% CO_2_. Live‐cell imaging was performed with a Leica confocal microscope using a 63× oil objective. Fluorescence intensity was analysed using ImageJ and presented as a series of curves in GraphPad Prism 9.5.0. High‐resolution imaging analysis of individual nucleoli was performed using a Zeiss Elyra 7 structured illumination microscopy (SIM) system with a 63× oil objective. Raw images were acquired and subsequently processed using structured illumination reconstruction algorithms to generate super‐resolution images. The resulting super‐resolution images were analysed using ImageJ software. Graphical representations of the data were generated, and line scan analysis was plotted using GraphPad Prism 9.5.0.

For nucleolar circularity analysis, nucleoli were identified by NPM1 immunofluorescence staining. Individual nucleoli were manually delineated in ImageJ based on the visible boundaries of NPM1‐enriched regions. Circularity was calculated using the ImageJ shape descriptor (Circularity = 4π × Area / Perimeter^2^), where values closer to 1 indicate a more circular shape. All measurements were performed using identical analysis parameters, and nucleoli were selected according to the same criteria across all conditions. The nucleolar circularity statistical analysis was plotted by Graphpad Prism 9.5.0 and analyzed by Two‐tailed Student's *t*‐test for two groups or One‐way analysis of variance (ANOVA) for more than two groups.

FRAP experiments were performed using a Leica STELLARIS 5 microscope equipped with a white light laser (WLL). Regions of interest (ROIs) were selected and photobleached using 35% laser power. Photobleaching was carried out for four cycles. Following photobleaching, fluorescence recovery within the bleached region was recorded. Fluorescence recovery curves were fitted using a single‐exponential recovery model implemented in the Leica confocal software. The immobile fraction was calculated based on the fitted recovery curves. Fluorescence intensities post‐bleach were normalized to the average intensity of two pre‐bleach images. Processed data were exported and plotted using GraphPad Prism 9.5.0.

### 4.6 Co‐IP, SDS‐PAGE, Native‐PAGE, and Western blotting (WB)

To detect the interaction between PCL2, EZH2, NPM1, and NPM1 with FBL via co‐IP, cells were lysed on ice for 30 min in WB and IP lysis buffer (Beyotime) containing 1% protease inhibitor cocktail (APExBIO) and 1 mM PMSF, treated with short‐lived sonication, and centrifuged for 10 min at 12 000 rpm. The supernatant was treated with protein A/G magnetic beads (Bimake) at 4°C for 1 h to eliminate nonspecific binding. The magnetic beads conjugated with the indicated antibody were used to incubate with the cell lysis. For the EGFP‐, FLAG‐tagged recombinant protein co‐IP assay, cells were collected and lysed as previously described, then the supernatant was treated with GFP‐ or FLAG‐ magnetic beads (Beijing LABLEAD Inc. Cat#PGM025, PFM025) at 4°C for 1 h to bind the recombinant proteins and their interacting proteins. The indicated proteins within the magnetic beads were detected with the corresponding antibodies for the WB assay.

To detect the indicated proteins in the denatured or native form, the cell lysates were mixed with SDS sample loading buffer for SDS‐PAGE or 5× Protein Native PAGE loading buffer (without heating) for Native‐PAGE. The gels were electrophoretically transferred onto a polyvinylidene fluoride membrane. The membranes were incubated with the indicated antibody and the corresponding secondary antibody. Images were captured and quantified using ImageJ software. The band intensity of proteins was normalised to the loading control (ACTB or histone H3), and the band intensity of phosphorylated proteins was normalised to the total proteins.

### 4.7 RNA Extraction, Reverse Transcription, and Quantitative Real‐Time Polymerase Chain Reaction (qRT‐PCR)

Total RNA was extracted with RNAiso Plus according to the recommended protocol. Complementary DNA was reverse‐transcribed using the ReverTra Ace qPCR RT Kit (TOYOBO). Quantitative real‐time PCR was performed on a LineGene9660 Real‐time PCR system (Bioer) using SYBR qPCR Mix (Tsingke). Primers for qRT‐PCR were designed according to published articles and PrimerBank [11, 66] and are listed in Table S2. The gene expression values relative to the housekeeping gene GAPDH were calculated by the Ct differences (ΔCt), and the fold change of the gene expression relative to the control sample was calculated by the difference between the ΔCt values (ΔΔCt).

### 4.8 Puromycylation Assay

HeLa, SiHa, and CaSki cells were seeded onto 6‐well plates and cultured overnight. Cells at approximately 40% confluence were transfected with shPCL2/shEZH2 or with a PCL2 overexpression plasmid. After 48 h, cells were cultured in medium containing 20 µg/mL puromycin for 20 min to label nascent peptide chains. Cells were lysed for SDS‐PAGE and WB using a specific anti‐puromycin antibody.

### 4.9 EU Staining

After transfection with PCL2‐FLAG, PCL2‐shRNA, or empty vectors, respectively, pre‐warmed culture medium containing 10 mM EU was added to the original cell culture system at a 1:1 volumetric ratio. Incubate the cells for an additional 5 h. Following incubation, harvest the cells and wash them three times with phosphate‐buffered saline (PBS). All subsequent steps were performed according to the recommended protocol. Cellular signals were acquired using a confocal laser scanning microscope. Quantitative analysis was conducted using ImageJ software, and statistical significance analyses were generated using GraphPad Prism software.

### 4.10 CCK8, EdU Staining Assays

For the CCK‐8 assay, the cells were cultured in 96‐well plates with a starting density of 1500 cells per well. According to the manual, cell proliferation was evaluated by the CCK‐8 kit (Meilun). For EdU staining, cells were seeded at the appropriate density in 6‐well plates and cultured overnight. EdU (Meilun) was added to the medium for an additional 0.5‐1 h of incubation. Cells were collected, stained, and processed according to the recommended protocol. The cell cycle was analysed by a flow cytometer.

### 4.11 Quantification and Statistical Analysis

Statistical analyses were performed using a two‐tailed Student's *t*‐test for comparisons between two groups. One‐way analysis of variance (ANOVA) was used for comparisons among three or more groups. For data that did not follow a normal distribution, the Mann‐Whitney U test or Kruskal‐Wallis test was applied. Statistical significance was defined as *, *P* < 0.05; **, *P* < 0.01 and ***, *P* < 0.001.

## Author Contributions

L. Zhu, J. Cao and X. Li conceived and planned the experiments. L. Zhu conducted the majority of experiments, with assistance from J. Cao, W. Wang, S. Chen, G. Zhu, Z. Wu, Y. Gu, J. Xiong, X. Zhang, H. Liu, and Y. Zhang. L. Zhu, J. Cao, and X. Li analysed the data. L. Zhu, J. Cao, and X. Li wrote the paper with input from all authors.

## Funding

This study was supported by the Natural Science Foundation of China (No. 31571321, 31900147), Natural Science Foundation of Shandong Province, China (No. ZR2022MH003, ZR2023MC091), and the Foundation of Qingdao Postdoctoral Innovation Project (QDBSH20230101019).

## Conflicts of Interest

The authors declare no conflicts of interest.

## Supporting information




**Supporting File**: advs76125‐sup‐0001‐SuppMat.docx.

## Data Availability

The data that support the findings of this study are available from the corresponding author upon reasonable request.
