# Supplementary material for: PRC2.1 Coordinates Peri‐Nucleolar H3K27me3‐Enriched Heterochromatin Organization and NPM1 Pentamerization to Maintain Nucleolar Integrity
Source: Adv Sci (Weinh). 2026 Jun 16:e19359. Online ahead of print. doi: 10.1002/advs.202519359 (PMC13336877; doi:10.1002/advs.202519359)
Supplement: Supplementary file 1 — Supporting File: advs76125‐sup‐0001‐SuppMat.docx. [file ADVS-9999-e19359-s001.docx]

Supporting Information

**PRC2.1 Coordinates Peri-nucleolar H3K27me3-enriched heterochromatin Organization and NPM1 Pentamerization to Maintain Nucleolar Integrity**

Lina Zhu, Wenqin Wang, Shuyun Chen, Gangyi Zhu, Zhen Wu, Yue Gu, Jingwen Xiong, Xu Zhang, Heng Liu, Kyoichi Isono, Youming Zhang, Jingjing Cao*, Xiangzhi Li*

**Supplementary Methods**

**Apoptosis assay**

Annexin V-FITC Apoptosis Detection Kit was used for analyzing the apoptosis rate according to the recommended protocol [1] via flow cytometer.

**Protein expression and purification**

EGFP-PCL2 was cloned into the pET-28a vector. Recombinant 6×His-EGFP-PCL2 was expressed in *Escherichia coli* BL21 and purified as previously described [2]. The purified protein was dialyzed overnight against storage buffer [25 mM Tris-HCl (pH 7.4), 150 mM NaCl]. The EGFP-PCL2 liquid droplets were prepared in the presence of the crowding agent 10% PEG-8000. The mixture (total protein concentrations are 5 μM, 10 μM, 20 μM) across a range of KCl concentration was visualized with a 100× oil immersion objective under Leica STELLARIS 5 microscope.

**Colony formation**

For colony formation assay, 1500 cells were seeded onto 6-well plates and cultured in normal growth medium for 2 weeks. Cells were then fixed and stained with 0.5% crystal violet (Beyotime) for 15 min, then washed with PBS for three times, and imaged.

**
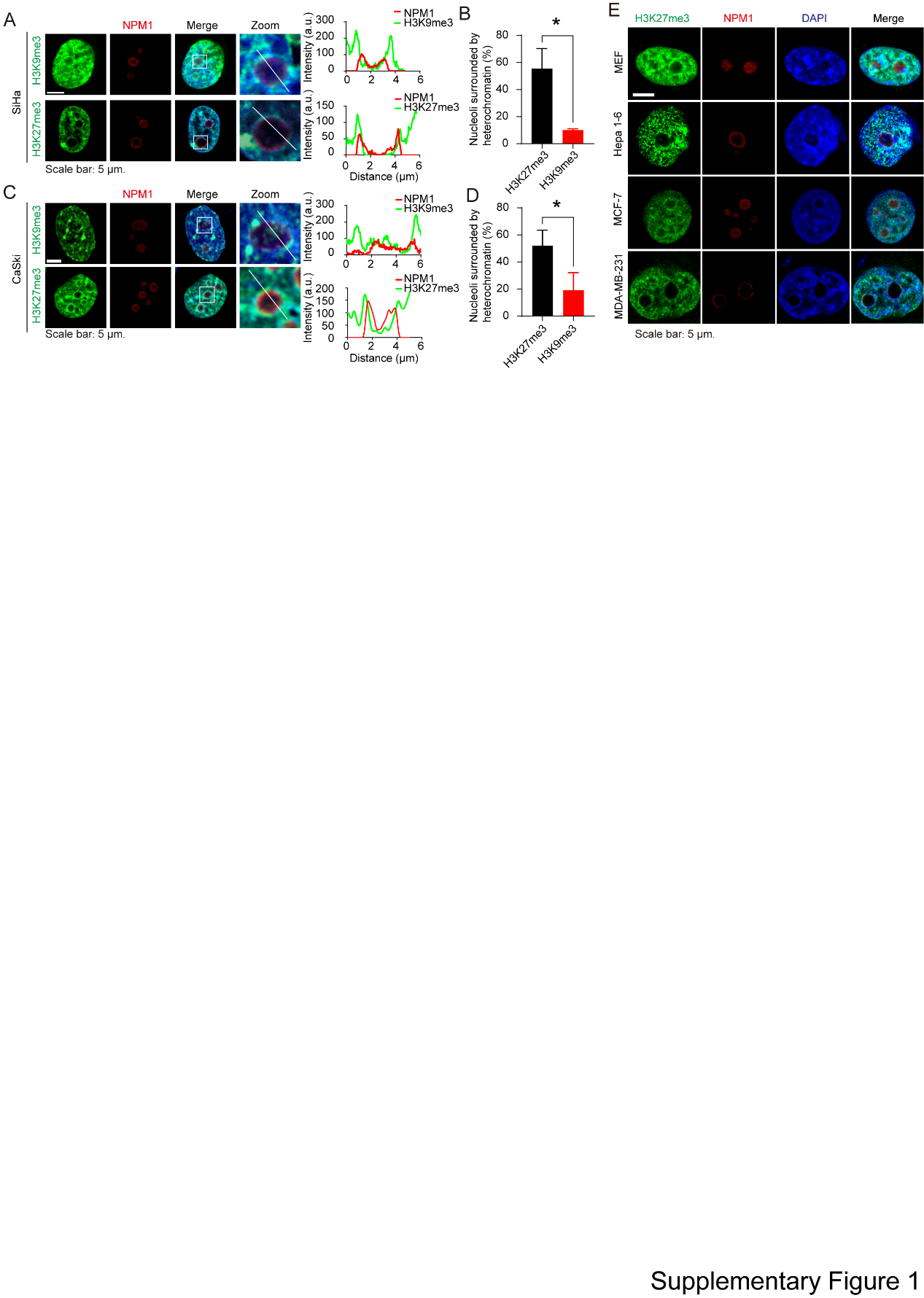
**

**Figure S1. H3K27me3 accumulates in peri-nucleolar regions.**

(A-D) Left panels: Subcellular localization of H3K27me3 or H3K9me3 (green), NPM1 (red), and DAPI (blue) in SiHa (A) and CaSki (C) cells is shown in the representative immunofluorescence (IF) images. Right panels: intensity profiles of NPM1, H3K27me3 and H3K9me3 correspond to the zoomed regions (white rectangle) indicated by white lines crossing the nucleolus in the left panels. Y-axis: intensity, arbitrary units (au); X-axis: distance (μm). Individual nucleoli from triplicate independent experiments in (A, C) were analyzed in (B, D). The nucleoli surrounded by ring-like structure of H3K9me3 or H3K27me3 were counted and presented as a percentage (%) of the total nucleoli in SiHa, and CaSki cells (the total nucleoli number, n>200). Statistical analysis was performed with two-tailed Student’s *t*-test. (E) Subcellular localization of H3K27me3 (green), NPM1 (red), and DAPI (blue) in MEF, Hepa 1-6, MCF-7, and MDA-MB-231 cells is shown in representative IF images. *, *P* < 0.05.


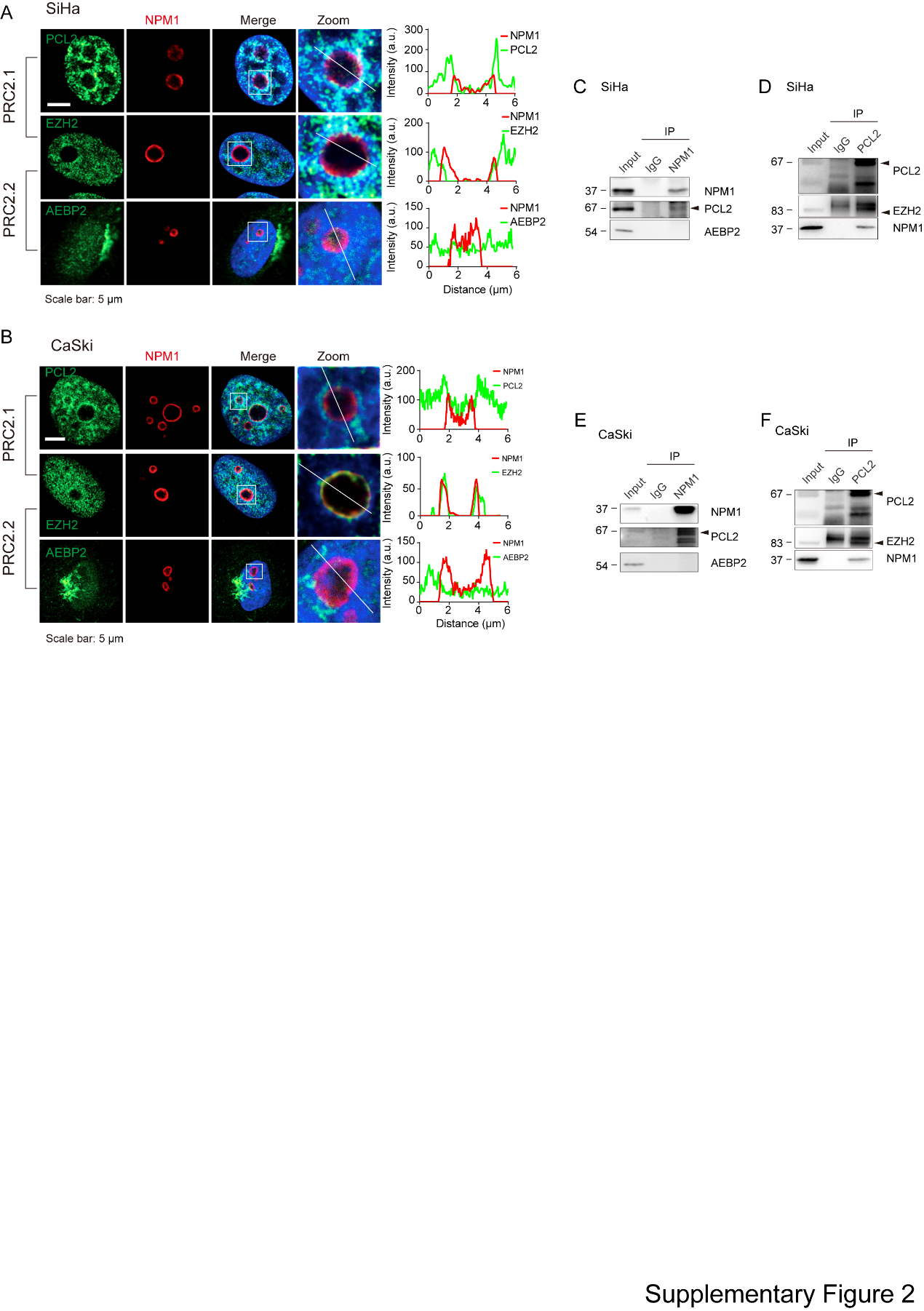


**Figure S2. PRC2.1 components colocalize and interact with NPM1.**

(A-F) Experiments in SiHa and CaSki cells were performed as described in Figure 1C- 1E.

**
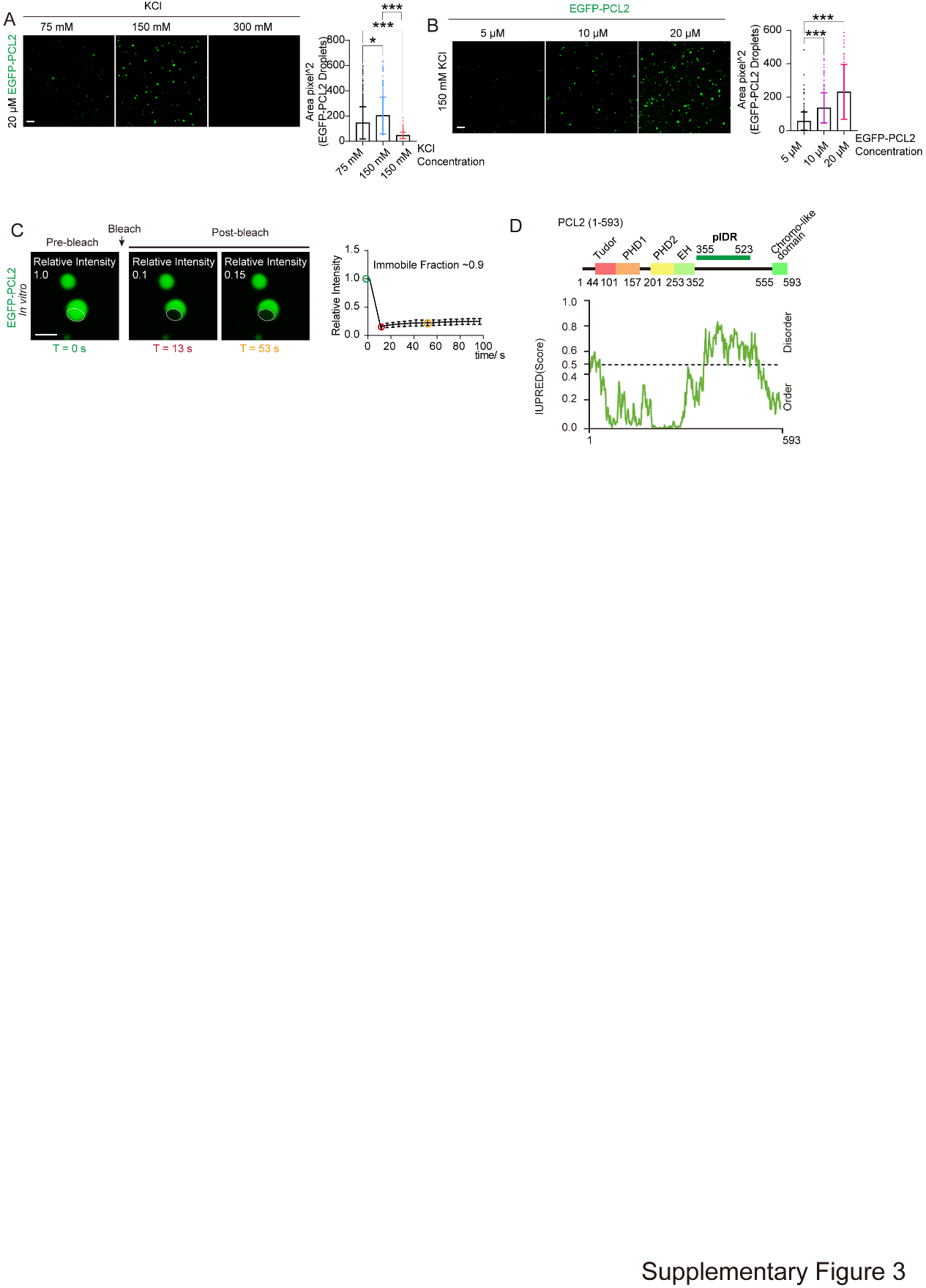
**

**Figure S3. PCL2 forms condensates both *in vivo* and *in vitro*.**

(A) Left panels: Fluorescence images of recombinant EGFP-PCL2 (20 μM) forming liquid-like droplets in buffer containing the indicated KCl concentrations (75, 150, 300 mM) and 10% PEG-8000. Right panels: Quantification of droplet size from the images shown in the left panels. Data were analyzed with ImageJ and plotted using GraphPad Prism. Scale bar, 10 μm. (B) Left panels: Fluorescence images of recombinant EGFP-PCL2 (5 μM, 10 μM, 20 μM) formed liquid-like droplets in buffer containing 150 mM KCl and 10% PEG-8000. Right panels: Quantification of droplet size from the images shown in the left panels. Data were analyzed with ImageJ and plotted by GraphPad Prism. Scale bar, 10 μm. (C) Left panels: The dynamic intensity of recombinant EGFP-PCL2 aggregates was analyzed using FRAP assay *in vitro*. A representative area is marked by a white circle. Foci were photobleached using 35% laser power for three cycles. Representative images of recombinant EGFP-PCL2 aggregates within the white circle before and after photobleaching are shown. Right panels: Recovery kinetics of EGFP-PCL2 aggregates from the left panels are shown. Data are from three FRAP events, and the values are presented as the mean ± SD. Scale bar, 2 μm. (D) Upper: Schematic diagram showing multiple domains in PCL2. The potential intrinsically disordered region (pIDR) of PCL2 was predicted using IUPred 2A. Bottom: Amino acid residues with a score greater than 0.5 were defined as disordered. This IDR is drawn as a green solid line in the upper panel. *, *P* < 0.05, **, *P* < 0.01; ***, *P* < 0.001.


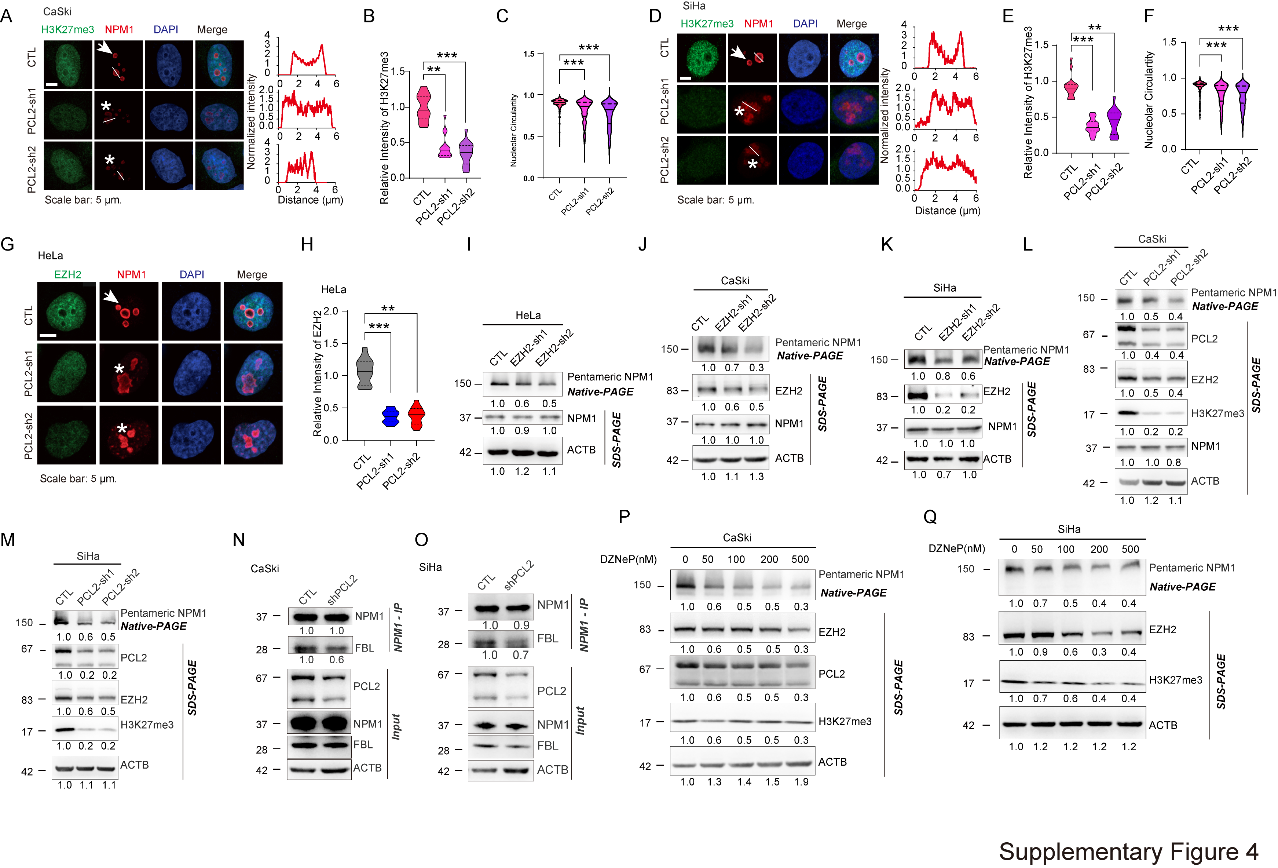


**Figure S4.** **PCL2 knockdown decreases EZH2 levels and disturbs nucleolar integrity.**

(A-F) The experiments in SiHa and CaSki cells were performed and analyzed according to the procedure in Figure 4A-4E. (G, H) PCL2-KD was performed using shRNA targeting PCL2 in HeLa cells, with an empty vector as a negative control (CTL). EZH2 (green), NPM1 (red), and DAPI (blue) were stained, and representative images are shown (G). Several images were acquired in the experiment (H), and the fluorescence intensity of EZH2 was measured and normalized to the CTL group. More than 35 cells in each group were collected for statistical analysis; the values are presented as the mean ± SD. (I-M) HeLa, CaSki, and SiHa cells were transfected with shRNA targeting PCL2 or EZH2. Cell lysates were submitted to Native-, SDS-PAGE, and the indicated proteins were detected with the corresponding antibodies. The gray value of each band was normalized to the CTL group. The fold change of the indicated protein was described under the bands. (N, O) CaSki and SiHa cells were transfected with the shPCL2 pool (PCL2-sh1 and PCL2-sh2), with co-IP performed to detect interaction between NPM1 and FBL. Cell lysates were immunoprecipitated with mouse anti-NPM1 antibody, and the intensity of NPM1 and FBL in the IP product was detected and normalized. (P, Q) Pentameric NPM1 in DZNeP and DMSO CaSki (P), SiHa (Q) cells was detected by Native-PAGE and immunoblotting. **, *P* < 0.01 and ***, *P* < 0.001.


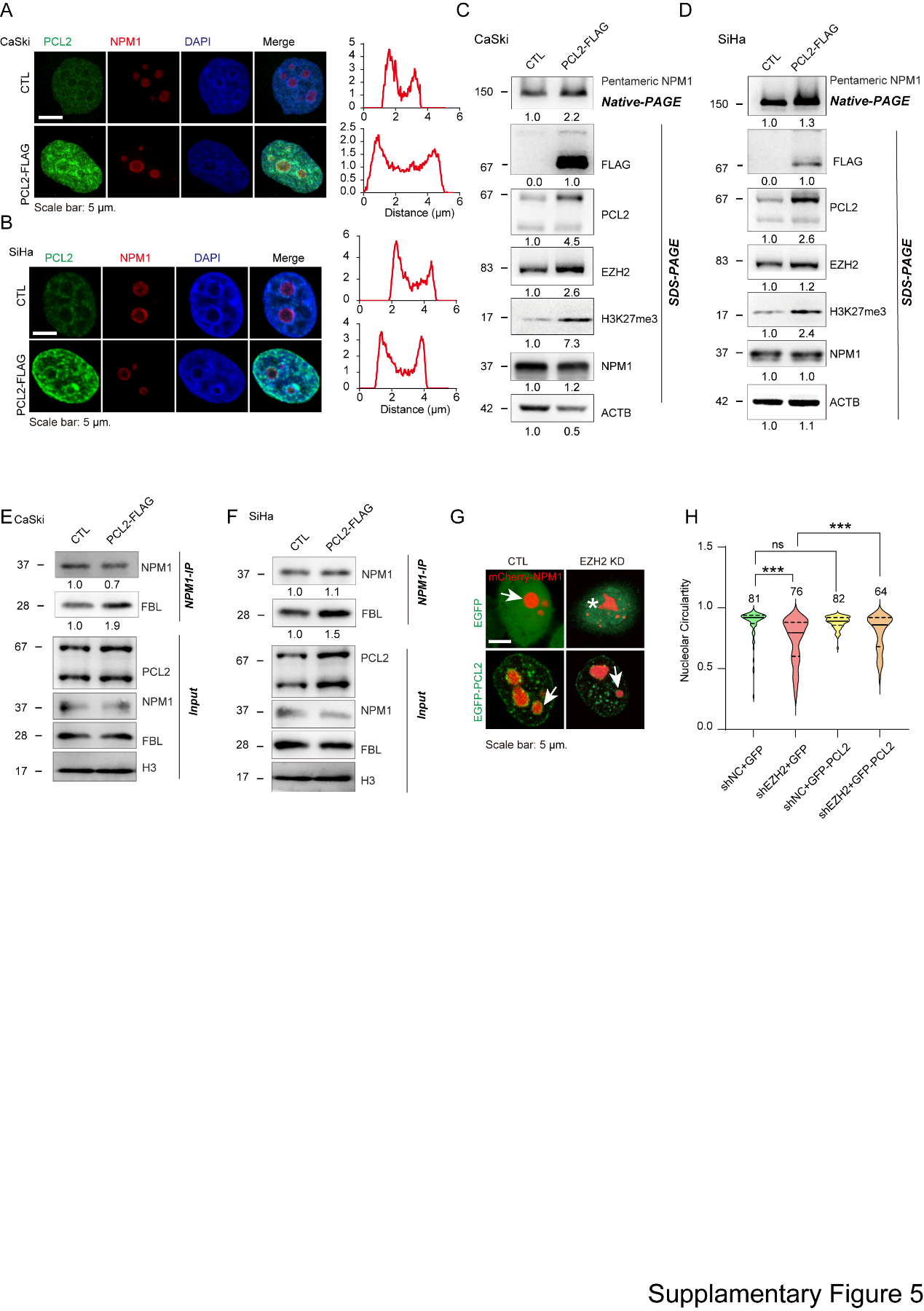


**Figure S5. Exogenous PCL2 increases H3K27me3 and pentameric NPM1 levels, and maintains nucleolar integrity.**

(A, B) Left panels: PCL2 overexpression was performed in CaSki (A), SiHa (B) cells. PCL2 (green), NPM1 (red), and DAPI (blue) were stained. NPM1 was used to indicate the nucleolar morphology. Scale bar, 5 μm. Right panels: The rim enrichment of NPM1 from the representative was shown. (C, D) Cell lysates from PCL2-FLAG and CTL groups were analyzed by Native-, SDS-PAGE and immunoblotting in CaSki (C) and SiHa (D) cells. The relative levels of the indicated proteins were normalized with the CTL group. Upper: pentameric NPM1. Bottom: FLAG, PCL2, EZH2, H3K27me3, NPM1 and ACTB. (E, F) Co-IP assay was performed in CaSki (E) and SiHa (F) cells overexpressing exogenous PCL2. Cells were lysed and immunoprecipitated using a mouse anti-NPM1 antibody. Immunoblotting was performed to detect the interaction between NPM1 and FBL. (G) Cells were infected with lentivirus to knock down EZH2 (EZH2-KD), followed by EGFP-PCL2/EGFP transfection (green). Lentivirus with the empty vector was used as a control (CTL). Nucleolar structure was marked with mCherry-NPM1 (red). Representative live-cell images are shown. The nucleolar morphology was analysed and classified into regular type and irregular type, indicated by white arrows and stars, respectively. (H) Multiple images from (G) were analyzed. Nucleolar circularity in each group was quantified, and the total number of analyzed nucleoli was greater than 60. Data were collected from three independent biological experiments. Statistical significance was analyzed using the Kruskal-Wallis test followed by Dunn’s multiple comparisons test.


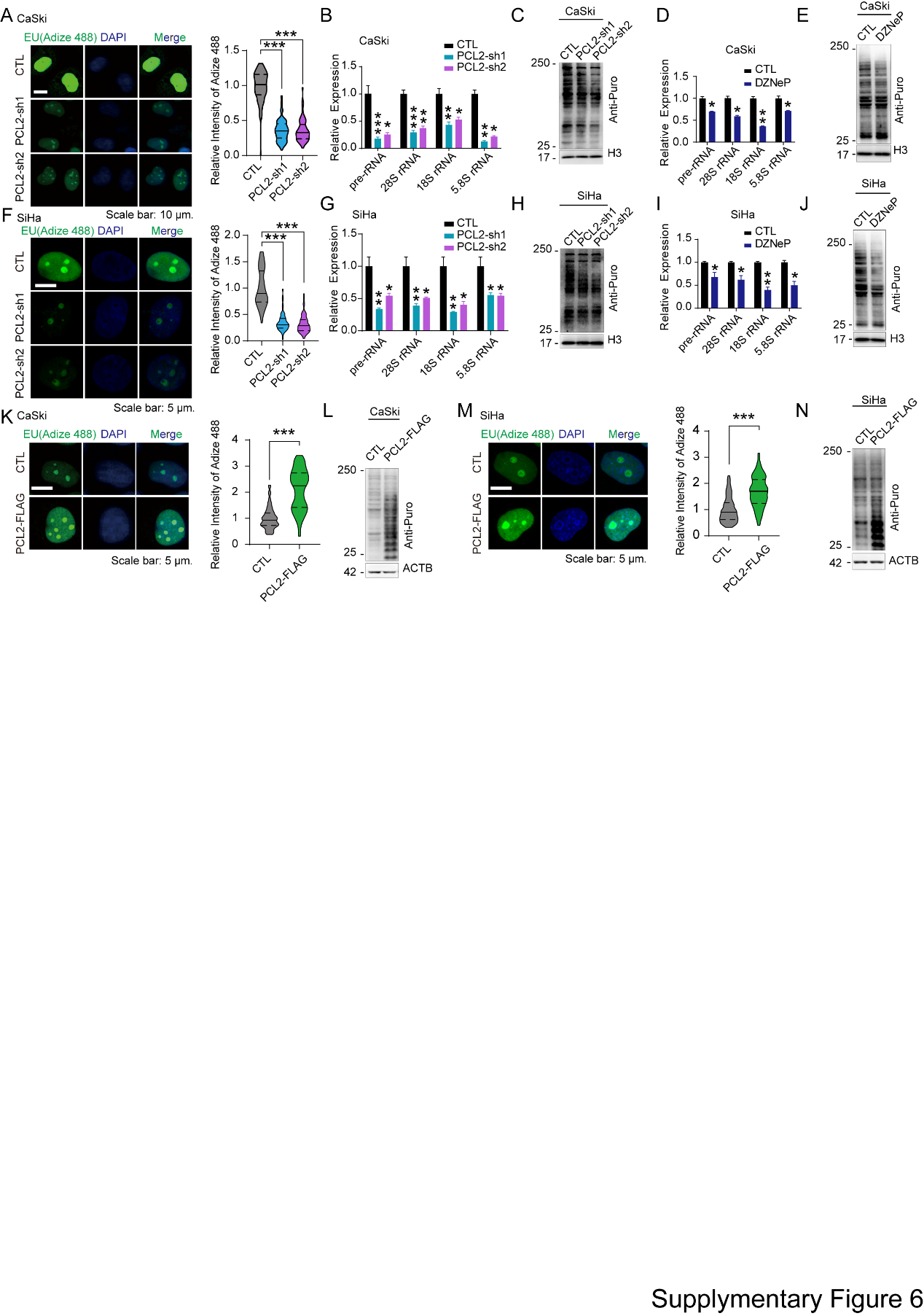


**Figure S6. PCL2 is critical for rRNA and global protein synthesis in CaSki and SiHa cells.**

(A-N) Experiments in SiHa and CaSki cells were performed as described in Figure 6. *, *P* < 0.05; **, *P* < 0.01; ***, *P* < 0.001.


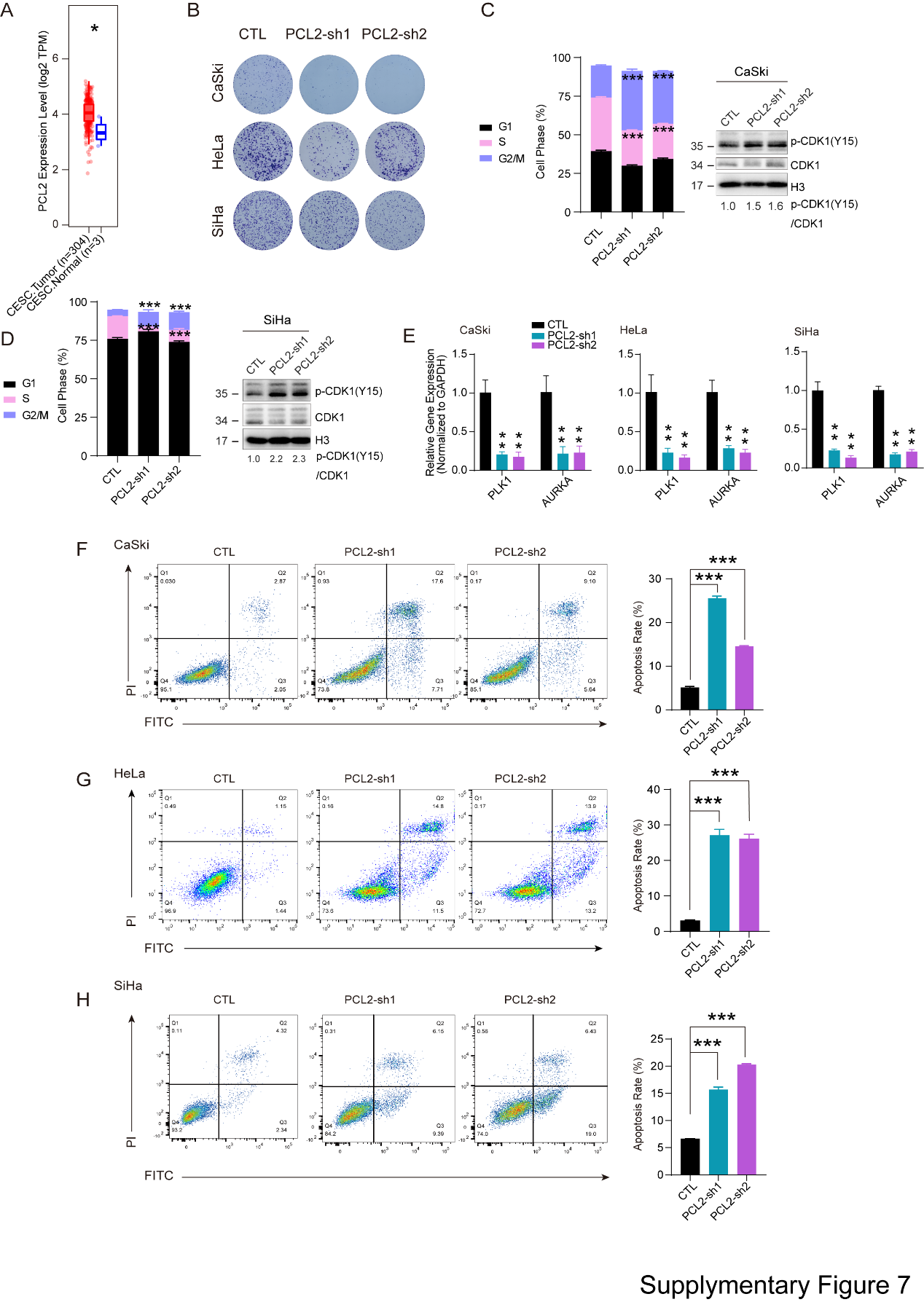


**Figure S7.** **Tumor tissues exhibit higher PCL2 expression than adjacent normal tissues, and PCL2 knockdown induces apoptosis in cancer cells.**

(A) Relative PCL2 expression level in patients, including tumor tissues (n = 304) and adjacent normal tissues (n = 3), was acquired from the Cancer Genome Atlas Cervical Squamous Cell Carcinoma and Endocervical Adenocarcinoma database (TCGA-CESC) and analyzed using Timer2.0. (B) Colony formation was performed to evaluate cell proliferation in PCL2-KD and CTL groups. Cell clusters were stained with crystal violet, and the number and size of blue dots were used to assess cell proliferation. (C, D) CaSki (C) and SiHa (D) cells were transfected with shRNA to knock down PCL2. At 48 hpt, EdU-labelled newly synthesized DNA was stained by Adize 555, while total DNA was stained by DAPI, followed detected by flow cytometry. The percentage of cells at G2/M in each group was analyzed and normalized to the CTL group in the left panel. The levels of p-CDK1(Y15) and CDK1 were detected with the corresponding antibodies and normalized to histone H3 in the right panel. The relative ratio of p-CDK1(Y15)/CDK1 is calculated and noted under the bands. (E) CaSki, HeLa, and SiHa cells were transfected with shRNA targeting PCL2, with an empty vector as a negative control (CTL). The transcription levels of the indicated genes, including *PLK1* and *AURKA,* were tested by RT-qPCR and normalized to *GAPDH*. The data are presented as the mean ± SD from three biological replicates. (F-H) Following by shRNA transfection for 48 h, CaSki, HeLa, and SiHa cells were stained with PI and FITC, and analyzed by flow cytometry (left). Apoptosis rate (%) were determined as the percentage of FITC-positive cells (right). Statistical significance was analyzed by one-way ANOVA followed by Dunnett’s post hoc test. ns, not significant; *, *P* < 0.05; **, *P* < 0.01; ***, *P* < 0.001.


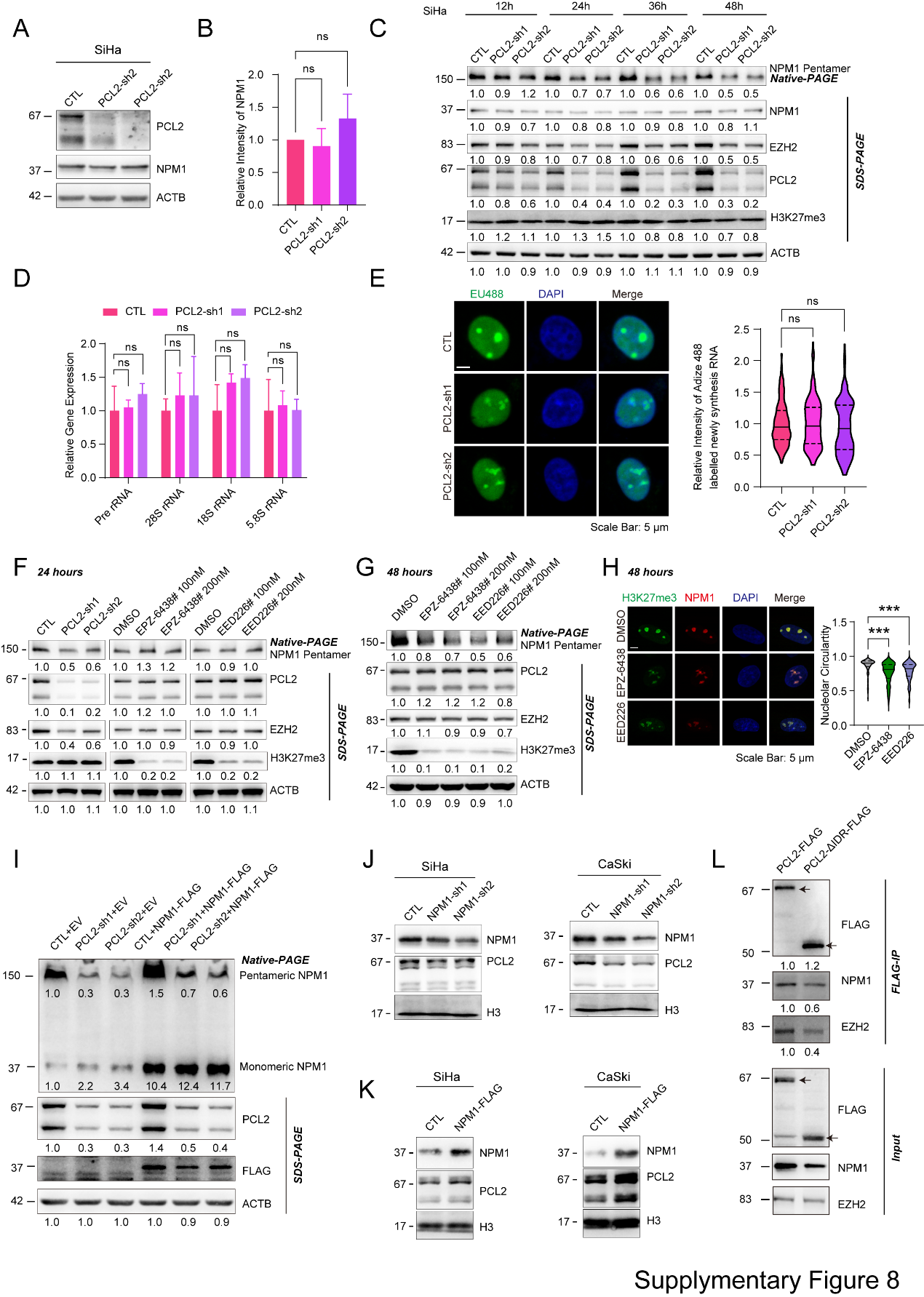


**Figure S8**. **Functional crosstalk between PCL2 and NPM1 in maintaining nucleolar homeostasis.**

(A, B) Total NPM1 level was detected by SDS-PAGE after PCL2 knockdown (A) in CaSki cells, and the relative level of total NPM1 was analyzed and plotted from three independent biological duplicates (B). (C) Pentameric NPM1 in shPCL2 and CTL cells was detected using Native-PAGE and immunoblotting at 12, 24, 36, 48 hours post-transfection. The level of PCL2, total NPM1 and EZH2 was detected in SDS-PAGE, with ACTB as loading control. (D) SiHa cells were transfected with shRNA to knock down PCL2. At 24 hpt, relative expression levels of pre- and mature rRNAs (28S/18S/5.8S), normalized to GAPDH, were measured by qRT-PCR. Data presented are means ± SD from three biological replicates. (E) At 24 hpt, EU-labelled newly synthesized RNAs were clicked and captured in siRNA-mediated PCL2 knockdown cells. Representative images are shown in the left panel, with relative intensity shown in the right panel. (F) Pentameric NPM1 in shPCL2, EPZ-6438, EED-226 treatment cells and CTL cells was detected using Native-PAGE and immunoblotting at 24 hours post-transfection and PRC2 inhibitor post-treatment. The level of PCL2, total NPM1 and EZH2 was detected using SDS-PAGE and immunoblotting, with ACTB as loading control. (G) Pentameric NPM1, PCL2, total NPM1 and EZH2 in EPZ-6438, EED-226 treatment cells and CTL cells were detected at 48 hours with PRC2 inhibitor post-treatment according to (F). (H) Left panel, subcellular localization of H3K27me3 (green), NPM1 (red), and DAPI (blue) in SiHa cells is shown in the representative IF images, following PCL2 or EZH2 knockdown. Relative fluorescence intensity profiles of NPM1 along the indicated white lines across the nucleolus are shown in the right panels. Nucleolar circularity in the left panels was quantified, and the total number of nucleoli in each group was greater than 100. Data were collected from three independent biological experiments. Statistical significance was determined using the Kruskal-Wallis test followed by Dunn’s multiple comparisons test. (H) CaSki cells were transfected with shRNA targeting PCL2, with a negative shRNA as control. At 12h post-transfection, the cells were subsequently transfected with exogenous NPM1-FLAG or an empty vector. After 48h, the levels of pentameric NPM1, PCL2, and overexpressed NPM1-FLAG were detected with the indicated antibodies, with ACTB as a loading control. (J, K) SiHa (left) and CaSki (right) cells were transfected with shRNA targeting NPM1 (J) or exogenous NPM1-FLAG (K), with the empty vector as a control. At 48h post-transfection, the expression levels of NPM1 and PCL2 were detected, and Histone H3 was used as a loading control. (L) Co-IP was performed in HeLa cells. Cells were transfected with plasmids expressing FLAG-tagged PCL2-WT/ΔIDR, and cell lysates were immunoprecipitated using FLAG-magnetic beads. Immunoblotting was performed to detect FLAG-tagged protein, endogenous EZH2, and NPM1. Scale bar, 5 μm. Statistical significance was determined by one-way ANOVA followed by Dunnett’s post hoc test. ns, not significant; *, *P* < 0.05; **, *P* < 0.01; ***, *P* < 0.001.

**
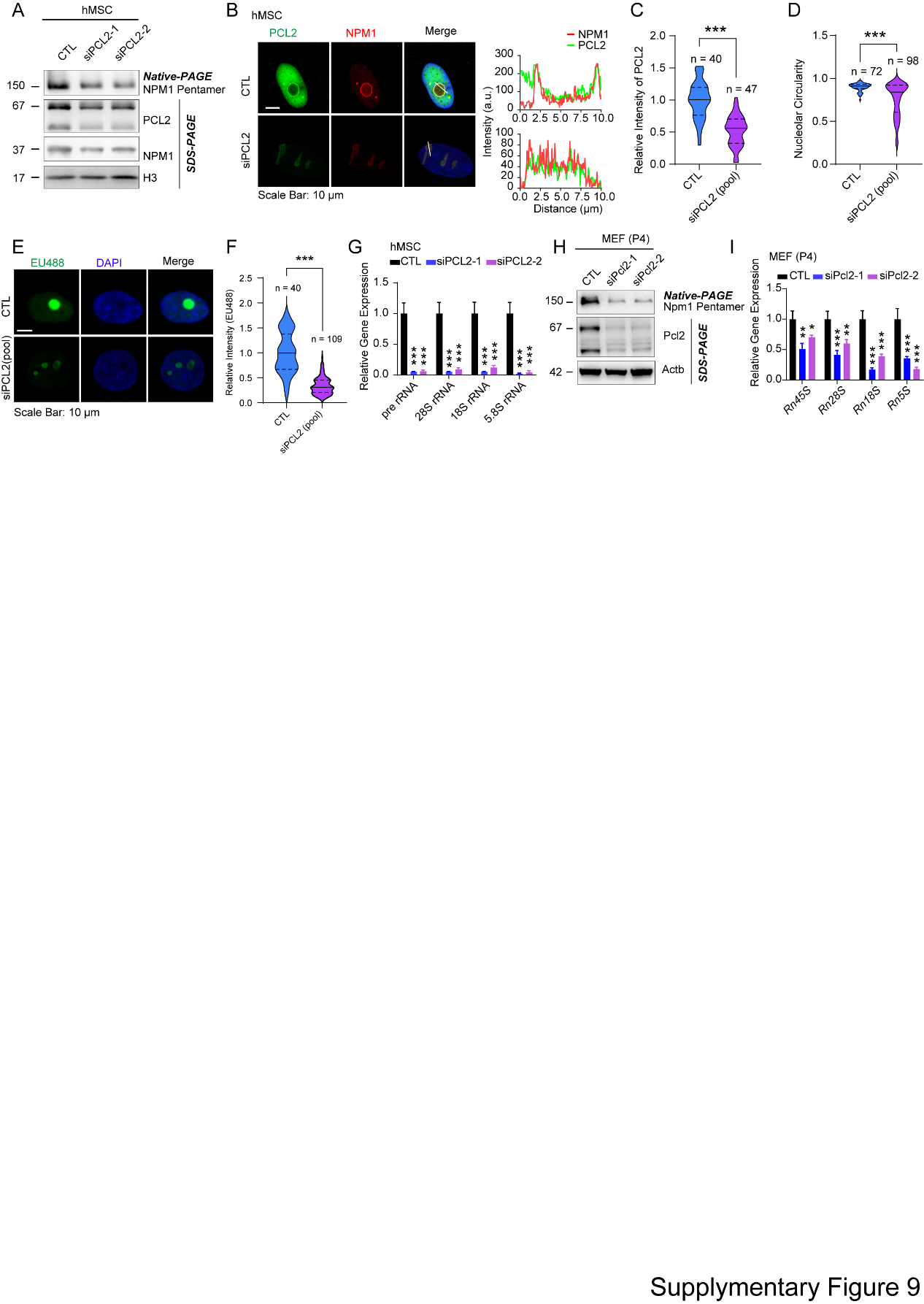
**

**Figure S9**. **PCL2 deficiency results in reduced pentameric NPM1 and rRNA in primary cells.**

(A) Pentameric NPM1 in siRNA-mediated PCL2 knockdown and negative control hMSC cells was analyzed by Native-PAGE and immunoblotting. (B) hMSC cells were transfected by siPCL2 pool (PCL2-si1 and PCL2-si2). PCL2 (green), NPM1 (red), and DAPI (blue) staining in CTL/siPCL2 cells. (C) The relative fluorescence intensity of PCL2 in the siPCL2 group was measured and normalized to the CTL group. Violin plots show the distribution of individual data points, with the central line indicating the median and the dashed lines indicating the interquartile range. Statistical significance was analyzed using the Mann-Whitney U test, and n means the total number of analyzed individual cells from three independent biological replicates. (D) Nucleolar circularity analysis based on (B) was performed and shown with violin plots, and n means the total number of analyzed nucleoli from three independent biological replicates. Statistical significance was analyzed using the Mann-Whitney U test. (E) EU-labelled newly synthesized RNAs were clicked and captured in siRNA-mediated PCL2-deficient cells. Representative images are shown in (E), with relative intensity shown in (F), and significance analysis with Mann-Whitney U test. (G) hMSC cells were transfected with siRNA to knock down PCL2. Relative expression levels of pre- and mature rRNAs (28S/18S/5.8S), normalized to ACTB, were measured by qRT-PCR. Data presented are means ± SD from three biological replicates, with significance analysis by ANOVA followed by Dunnett’s post hoc test. (H) Pentameric NPM1, pre- and mature- rRNA (I,) in MEF cells were detected, respectively, as described above. Statistical significance was analyzed by one-way ANOVA followed by Dunnett’s post hoc test. *, *P* < 0.05; **, *P* <0.01; ***, *P* < 0.001.


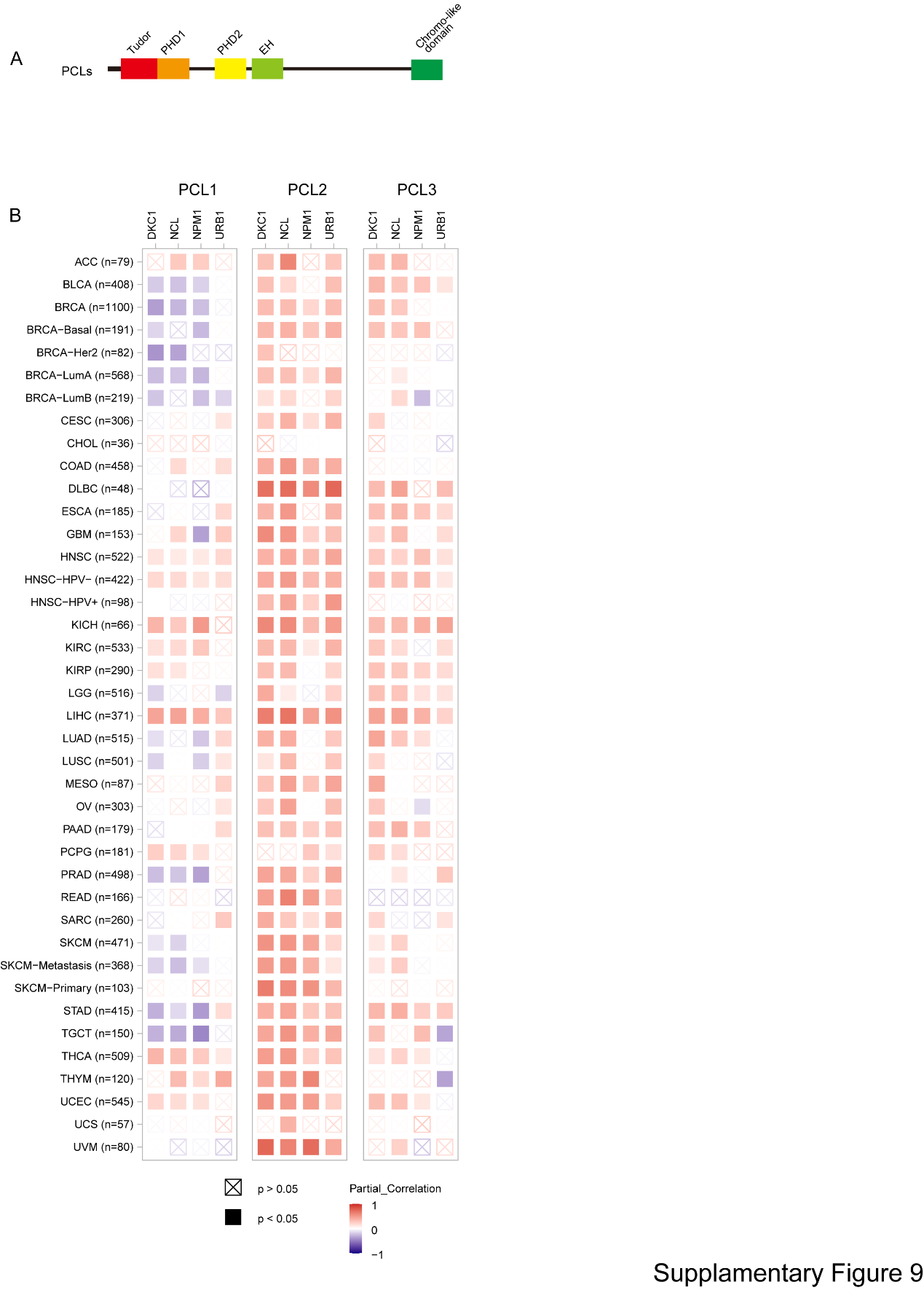


**Figure S10. Correlation analysis between PCLs and the nucleolus protein-coding genes.**

(A) A pattern diagram of PCLs is shown, in which the Tudor domain, PHD, EH and chromo-like domain have been identified. (B) The correlation between PCLs genes (PCL1/PCL2/PCL3) and nucleolar protein-coding genes (DKC1/NCL/NPM1/URB1) was analyzed using the Cancer Genome Atlas (TCGA) program and Timer 2.0 database. Red and purple boxes indicate positive and negative correlations, respectively. The box with diagonal lines indicated not significant (*P* > 0.05), while solid box indicated significant (*P* < 0.05).

**Table S1. PCR primers used for plasmid construction.**

| Oligo name | Sequence (5’-3’) |
| --- | --- |
| LV-PCL2-Gi-F | TAGCGCTACCGGACTCAGATCATGAGAGACTCTACAGGGGCAG |
| PCL2-FLAG | TCACTTATCGTCGTCATCCTTGTAATCGGATGCAGTTGCTCCTTCCCAT |
| LV-FLAG-Gi-R | CTCCCCTACCCGGTAGAATTATTCACTTATCGTCGTCATCCTTGTAATC |
| eC1-PCL2-Gi-F | CGAGCTCAAGCTTCGATGAGAGACTCTACAGGGGCAG |
| eC1-PCL2-Gi-R | TCAGTTATCTAGATCCGCTAGGATGCAGTTGCTCCTTCCCATTC |
| mC1-NPM1-Gi-F | GTACAAGTCCGGACTCAATATGGAAGATTCGATGGACATGGACA |
| mC1-NPM1-Gi-R | CCGGGCCCGCGGTACTTAAAGAGACTTCCTCCACTGCCAGAG |
| NPM1-F | ATGCCAGTCCCCGAGCGCCCTGC |
| NPM1-R | GGCGGGCTGCTCCAGCTCCAT |
| FLAG-NPM1-R | TTATCGTCGTCATCCTTGTAATCGGCGGGCTGCTCCAGCTCCAT |
| 3.1-FLAG-Gi-R | GGTTTAAACGGGCCCTTTATTACTTATCGTCGTCATCCTTGTAATC |
| NPM1-Gi-F | AGCTTGGTACCGAGCTCGATGCCAGTCCCCGAGCGCCC |
| FLAG-NPM1-N-R | CTTATCGTCGTCATCCTTGTAATCTTCTACAGAACTAGGTCCTTTTGGTG |
| PCL2-ΔIDR-F | CGTGTTCCTCCTGTGCCAGATTATCAGTTTGATGAACTCAACA |
| PCL2-ΔIDR-R | TCATCAAACTGATAATCTGGCACAGGAGGAACACGA |
| eC1-PCL2-IDR-Gi-F | CGAGCTCAAGCTTCGCCAAATGTGGCTTTCAAAGCAGAGA |
| eC1-PCL2-IDR-Gi-R | TCAGTTATCTAGATCCGGTGCTATTCTTTGCCTTCATCATCTTTTACAATTTCTGAGT |
| CAG-PCL2-Gi-F | CGAGCTGTACAAGAGCATGAGAGACTCTACAGGGGCAG |
| CAG-FLAG-Gi-R | TTTTGGCAGAGGGAAAAACTACTTATCGTCGTCATCCTTGTAATC |
| PCL2-sh1-F | CCGGTCCCAATGAAATGGTTATATGCTCGAGCATATAACCATTTCATTGGGATTTTTG |
| PCL2-sh1-R | AATTCAAAAATCCCAATGAAATGGTTATATGCTCGAGCATATAACCATTTCATTGGGA |
| PCL2-sh2-F | CCGGCCATTACAGTGGGTAGATATACTCGAGTATATCTACCCACTGTAATGGTTTTTG |
| PCL2-sh2-R | AATTCAAAAACCATTACAGTGGGTAGATATACTCGAGTATATCTACCCACTGTAATGG |
| EZH2-sh1-F | CCGGTATGATGGTTAACGGTGATCACTCGAGTGATCACCGTTAACCATCATATTTTTG |
| EZH2-sh1-R | AATTCAAAAATATGATGGTTAACGGTGATCACTCGAGTGATCACCGTTAACCTCATA |
| EZH2-sh2-F | CCGGCCCAACATAGATGGACCAAATCTCGAGATTTGGTCCATCTATGTTGGGTTTTTG |
| EZH2-sh2-R | AATTCAAAAACCCAACATAGATGGACCAAATCTCGAGATTTGGTCCATCTATGTTGGG |

**Table S2. PCR primers used for qRT-PCR.**

| Oligo name | Sequence (5’-3’) |
| --- | --- |
| Pre-rRNA-F | GCCTTCTCTAGCGATCTGAGAG |
| Pre-rRNA-R | CCATAACGGAGGCAGAGACA |
| 28S rRNA-F | GCGGGTAAACGGCGGGAGTA |
| 28S rRNA-R | TTGGCTGTGGTTTCGCTGGAT |
| 18S rRNA-F | TCCTTTGGTCGCTCGCTCCT |
| 18S rRNA-R | GATCTGATAAATGCACGCATCCC |
| 5.8S rRNA-F | ACTCGGCTCGTGCGTC |
| 5.8S rRNA-R | GCGACGCTCAGACAGG |
| *GAPDH*-F | GGTCACCAGGGCTGCTTTTA |
| *GAPDH*-R | TTCCCGTTCTCAGCCTTGAC |
| *PLK1*-F | CCTGCACCGAAACCGAGTTAT |
| *PLK1*-F | CCGTCATATTCGACTTTGGTTGC |
| *AURKA-*F | GGAATATGCACCACTTGGAACA |
| *AURKA-*R | TAAGACAGGGCATTTGCCAAT |

**Supplementary References**

1. D. Li, Y. Yang, B. Chen, et al., “MOF Regulates TNK2 Transcription Expression to Promote Cell Proliferation in Thyroid Cancer,” *Front Pharmacol* 11, no. (2020): 607605, <https://doi.org/10.3389/fphar.2020.607605>.

2. S. Huang, X. Mo, J. Wang, et al., “α-Synuclein phase separation and amyloid aggregation are modulated by C-terminal truncations,” *FEBS Lett* 596, no. 11 (2022): 1388-1400, <https://doi.org/10.1002/1873-3468.14361>.
